# Electronic measures of movement impairment, repositioning, and posture in people with and without neck pain—a systematic review

**DOI:** 10.1186/s13643-019-1125-2

**Published:** 2019-08-27

**Authors:** Bue Bonderup Hesby, Jan Hartvigsen, Hanne Rasmussen, Per Kjaer

**Affiliations:** 1 Department of Sports Science and Clinical Biomechanics, University of Southern Denmark, Campusvej 55, DK-5230 Odense M, Denmark; 2 Nordic Institute of Chiropractic and Clinical Biomechanics, Campusvej 55, DK-5230 Odense M, Denmark; 3 Slagelse Hospital, Region Zealand, Ingemannsvej 18, DK-4200 Slagelse, Denmark; 4 Health Sciences Research Centre, UCL University College, Niels Bohrs Allé 1, DK-5230 Odense, Denmark

**Keywords:** Neck pain, Range of motion, Motor control, Posture, Kinaesthetic, Kinematics

## Abstract

**Background:**

Neck pain is a major public health problem. Our objective was to describe differences in measures of movement and posture between people with and without neck pain.

**Methods:**

PubMed and Embase were searched before 15 February 2019 for studies comparing people with neck pain with controls using electronic measurements of neck movement and/or posture. Data were extracted on participants, device, test methods, active range of motion (RoM) and quality of motion, joint positioning sense, and posture. Study quality was assessed using the quality assessment of studies of diagnostic accuracy included in systematic reviews (QUADAS) and Guidelines for Reporting Reliability and Agreement Studies (GRRAS) guidelines.

**Results:**

Thirty-six studies were included: 24 studies included measurement of active RoM, 15 quality of motion, 12 joint positioning sense, and 5 cervical spine posture. Measurements and test methods were heterogeneous. The reporting of study populations and methods were poor, whereas devices and statistics were well described. All studies on RoM showed reduced active RoM in people with neck pain when compared with controls, 5 of 10 studies reported reduced movement speed for people with neck pain, and 5 of 9 studies reported significantly greater joint positioning error for people with neck pain compared with controls. Due to heterogeneous test parameters and methods, no conclusion regarding differences in conjunct motion, tracking a motion pattern, and measures of posture could be drawn.

**Conclusions:**

People with neck pain appear to have reduced active RoM, movement speed, and head repositioning accuracy when compared with controls. However, quality of reviewed studies was low and better descriptions of participants and methods are required before firm conclusions can be drawn.

**Electronic supplementary material:**

The online version of this article (10.1186/s13643-019-1125-2) contains supplementary material, which is available to authorized users.

## Background

Neck pain is a common condition with a reported point prevalence of between 0.4 and 41.5% and a lifetime prevalence ranging from 14.2 to 71.0%, depending on its definition [1–3]. Neck pain is ranked as the fourth highest contributor to years lived with disability [4]. In Denmark, 6% of all visits to general practitioners and 23% of all visits to chiropractors or physiotherapist are due to neck pain [5]. More than 300 definitions of neck pain have been used in the epidemiological literature [6–12]. In 2009, the Joint Decade 2000–2010 Task Force on neck pain introduced a conceptual model of neck pain, defining neck pain as pain or discomfort between the superior nuchal line and the spine of the scapula [6]. Treatment approaches such as acupuncture, patient education, multidisciplinary rehabilitation, joint mobilisation, manipulation, and exercise have been shown to be effective treatments, but effect sizes have been small to moderate [7–13], and there is no clear evidence for any treatment being superior to another. One way to potentially improve the effect of treatment could be to target interventions to specific impairments that clearly discriminate between different types of patients with neck pain or between people with and without neck pain [14]. Existing classification systems build on pain distribution and neurological findings [15], and severity and impact of neck pain [16, 17], whereas only one system of targeting treatment has been suggested [18]. However, none of these systems has been rigorously tested for its ability to clearly distinguish between people with and without neck pain.

Exercise treatment is widely used and has the ability to target specific impairments of the neck [19] or limit potential harmful postures such as carrying the head in a forward position [19]. Other parameters such as active range of motion [20–25], neck movement speed [26], conjunct motion [27], smoothness of motion [25, 28], and kinaesthetic sense [25, 29–35] have been used to guide how exercises are delivered and performed in individual patients. Assessment of these factors requires that the measurements are reliable and valid in order to correctly guide interventions. Often, these measurements were obtained using electronic devises capable of continuous measurements or movement impairments such as impaired joint position sense. To our knowledge, only one review has addressed movement impairments (joint positioning sense) in people with neck pain [36]. Therefore, there is a need for an overview of the different movement impairments in the neck pain population, measured with electronic devices, in order to provide clinicians and researchers with state-of-the-art knowledge about electronic measurements of neck impairments; the reliability and diagnostic value of these measures, considering the technology and practical application of the movement test; and the type of neck patients.

The overall aim of this systematic review was to determine whether people with neck pain have different movement patterns when compared with people without neck pain. Firstly, we summarised the electronic devices used, the measurement methods, and the definitions of people with and without neck pain. Secondly, we compared electronic measurements of active range of motion, quality of neck motion, joint repositioning accuracy, and posture in people with and without neck pain.

## Methods

### Study design

This study was a systematic literature review based on criteria adapted from Cochrane diagnostic studies [37] and reported according to the Preferred Reporting Items for Systematic reviews and Meta-Analyses (PRISMA) guidelines [38] (Additional file 1).

### Setting

This study was conducted at the Department of Sports Science and Clinical Biomechanics on University of Southern Denmark as part of the welfare tech project ‘patient@home’.

### Search strategy

We identified relevant studies from the databases PubMed and Embase. The search strategy was tailored with the assistance of an experienced research librarian. We limited the search to include only publications in English or Danish published between 1 January 2004 and 15 February 2019. The reference lists of all included papers were closely scrutinised for eligible studies. For the full search strategy, see Additional file 2.

### Inclusion criteria

We included cross-sectional studies where the case population was judged to have non-specific neck pain, whiplash-associated disorder (WAD), cervical radiculopathy, or acute, sub-acute, or chronic neck pain of any duration. Furthermore, the study had to report at least one electronic measure of a movement impairment, joint position sense, or posture. The inclusion and exclusion criteria are specified in Table 1.

**Table 1:** Inclusion and exclusion criteria used for selecting studies

	Inclusion criteria	Exclusion criteria
1) Participant	Cervical radiculopathy	Congenital malformations
	Whiplash-associated disorders	Any kind of neck surgery
	Non-specific neck pain	No controls without neck pain
	Acute, sub-acute, and chronic neck pain of all durations and severity	Younger than 18 years
		Neck pain caused by fractures, inflammatory joint disease, connective tissue diseases, infection, or tumours
2) Type of measurements	Active range of motion	
	Neck posture	
	Movement speed	
	Acceleration	
	Jerk (smoothness of motion)	
	Head repositioning accuracy	
	Kinematic	
	Kinaesthetic	
Device		Primary measurement obtained with MRI or X-ray
		Non-electronic measurement device
		No description of the measurement instrument
		No presentation of result for each group
3) Language	Danish or English	

### Selection of studies

The results from the literature searches were imported into EndNote©, and duplicates were removed. Three authors (BBH, HR, and PK) were involved in the screening process. Three authors (BBH and HR/PK) independently screened the titles and abstracts for relevance. If it was not possible to decide from title and abstract, a full-text screening was performed. In case of disagreement, a third author (PK/JH) was consulted.

### Data extraction

BBH, PK, and JH did the data extraction independently. This included information about study population, testing circumstances, and test device. The results from the impairment measures were extracted, including active range of motion, movement speed, acceleration, jerk, head repositioning accuracy, and posture.

### Quality assessment

The quality of the included studies was assessed using a purposeful tailoring of the quality assessment of studies of diagnostic accuracy included in systematic reviews (QUADAS) and Guidelines for Reporting Reliability and Agreement Studies (GRRAS) guidelines [39, 40] where we remodelled the element relating to a reference standard and questions about case-control design. We piloted the modified tool using articles that we had excluded from the review.

Quality assessment was done in two sets where one half of the included studies were evaluated by BBH and PK and the second half by BBH and JH. Disagreements between the authors were discussed and consensus sought, and continued disagreements were then resolved by JH for the first half and PK for the second half.

### Data reporting and analysis

Agreement in selecting studies and rating risk of bias were determined using Cohen’s kappa [41]. The results, including risk of bias, were grouped by type of measurement and reported for subgroups of different types of neck pain. For comparable measures and homogeneous studies, the results were presented as forest plots. For measures where studies were too heterogeneous for that, data were narratively summarised.

## Results

### Description of included studies

**Fig. 1:**
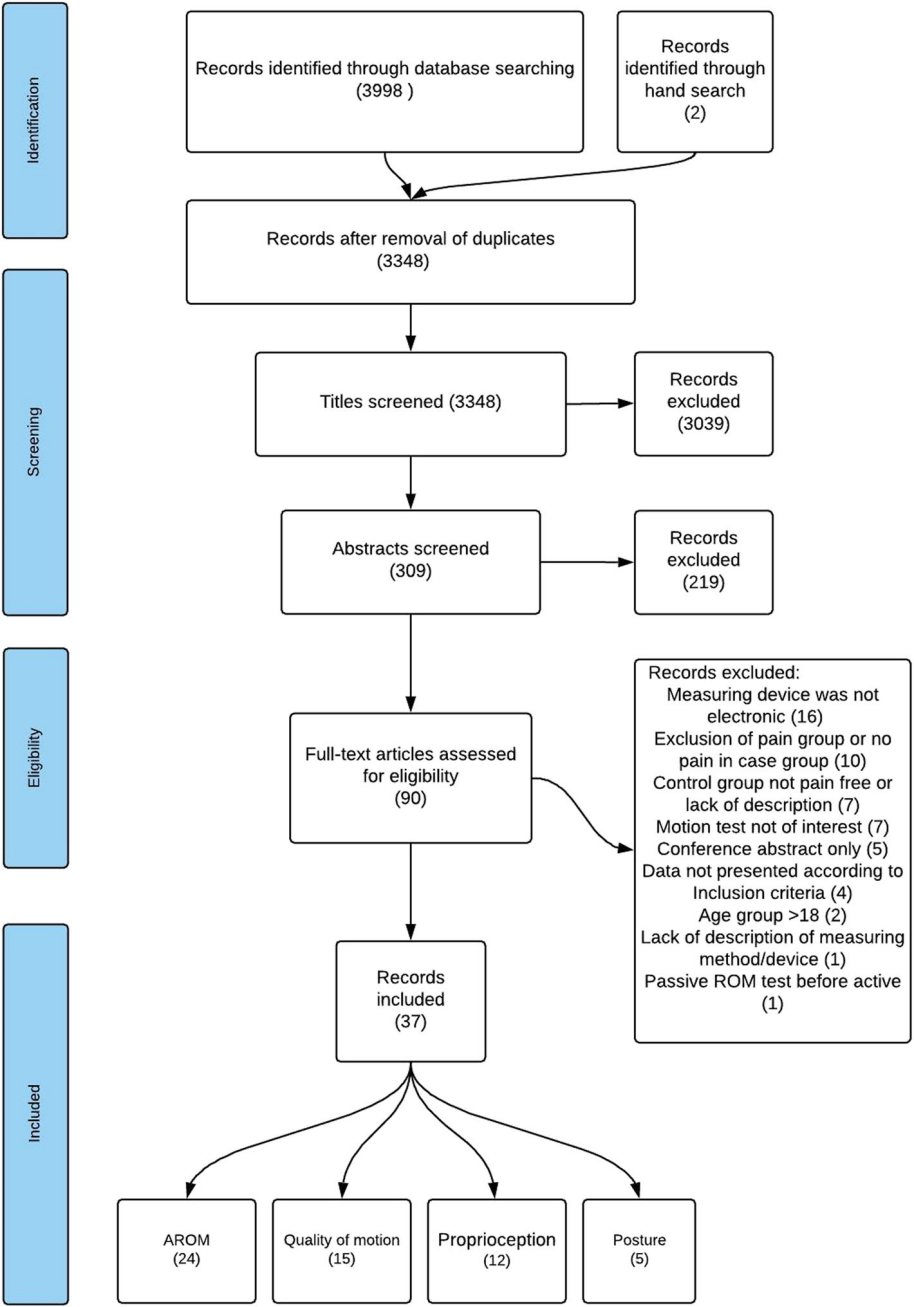
PRISMA flow diagram for inclusion of studies

The literature search was conducted on 5 November 2014, and updated on 19 September 2017 and again on 15 February 2019. We identified 3348 unique studies after excluding 652 duplicates. A total of 90 papers were retrieved in full text of which 53 were excluded (see Additional file 3 for a list of excluded studies). An overview of the selection process and reasons for exclusion are shown in Fig. 1. We identified a total of 37 papers reporting on 36 studies [21, 27, 29, 30, 34, 42–67]. These studies had case populations ranging between 7 and 120 participants with an average of 33 participants, and control populations ranging between 11 and 150 with an average of 35 participants. In 27 studies [22, 28, 30, 31, 35, 43, 45–51, 54–60, 62–64, 66, 69–71, 74], patients with neck pain were described as chronic or with pain duration of more than 3 months. In six studies [43, 51, 52, 59, 60], there was no description of pain duration. Definition of chronicity varied from no specification [29], duration of pain longer than 6 weeks [44], to neck pain lasting more than 2 years [30].

In 12 studies, patients with WAD defined as Québec Task Force grades type I–III [68] were included [27, 34, 42, 45, 46, 48–50, 53, 57, 66]. A total of 15 studies included people with non-specific neck pain labelled idiopathic neck pain [47, 71], non-specific neck pain [54, 67], no traumatic neck pain [66], myofascial neck pain [60], unilateral posterior neck pain [57], insidious neck pain [58], neck or shoulder disorder [44], or simply neck pain [47]. One study [48] defined postural neck pain as a pain in the neck aggravated by postural load and relieved with postural modification.

### Quality of included studies

The initial inter-rater reliability of the risk of bias assessment between BBH and PK had an agreement of 76.4% and a kappa score of 0.59. Between BBH and JH, the agreement was 60.6% with a kappa score of 0.38. The total agreement was 68.5% with a kappa score of 0.48. After discussion, consensus was reached for all items.

Most of the studies had insufficient description of sample size, study population, characteristics of raters, and blinding of raters for clinical information and previous findings. In contrast, the descriptions of measurement devices, tests, recording methods, and statistical analyses were generally adequately reported. Detailed results of the risk of bias assessments are presented in Table 2.

**Table 2:**
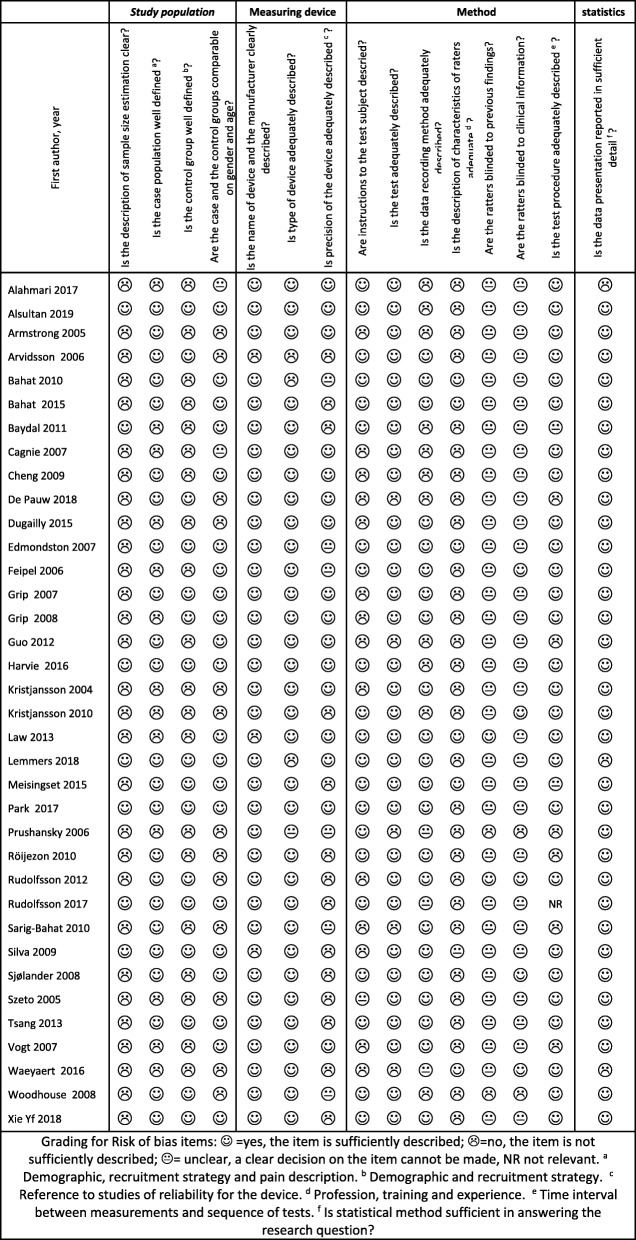
Assessment of the quality of the include studies

### Range of motion measures

**Fig. 2:**
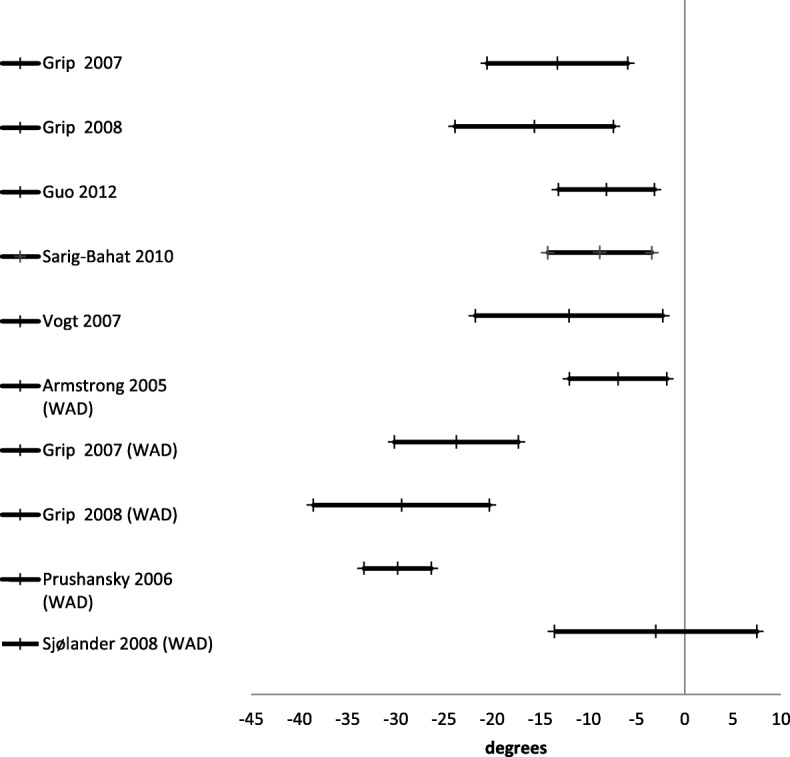
Right rotation. Mean difference in neck right rotation between people with neck pain and healthy controls

**Fig. 3:**
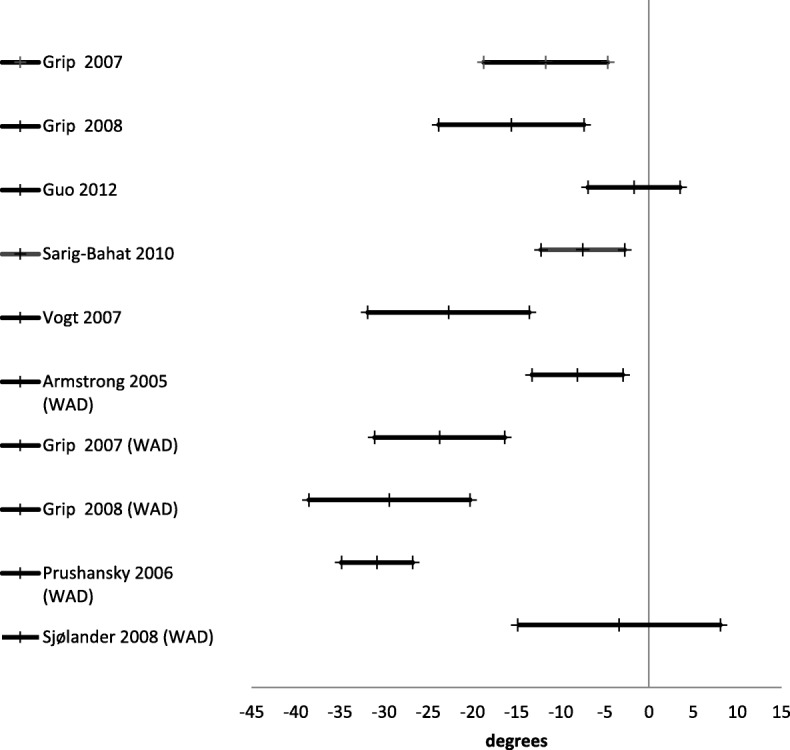
Left rotation. Mean difference in neck left rotation between people with neck pain and healthy controls

**Fig. 4:**
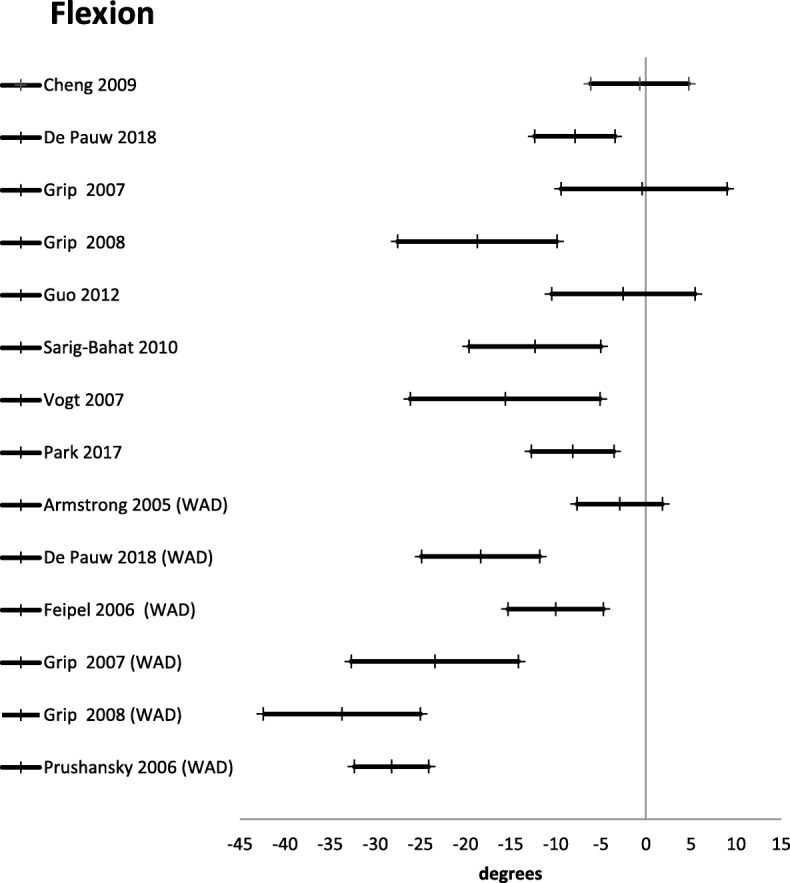
Flexion. Mean difference in neck flexion between people with neck pain and healthy controls

**Fig. 5:**
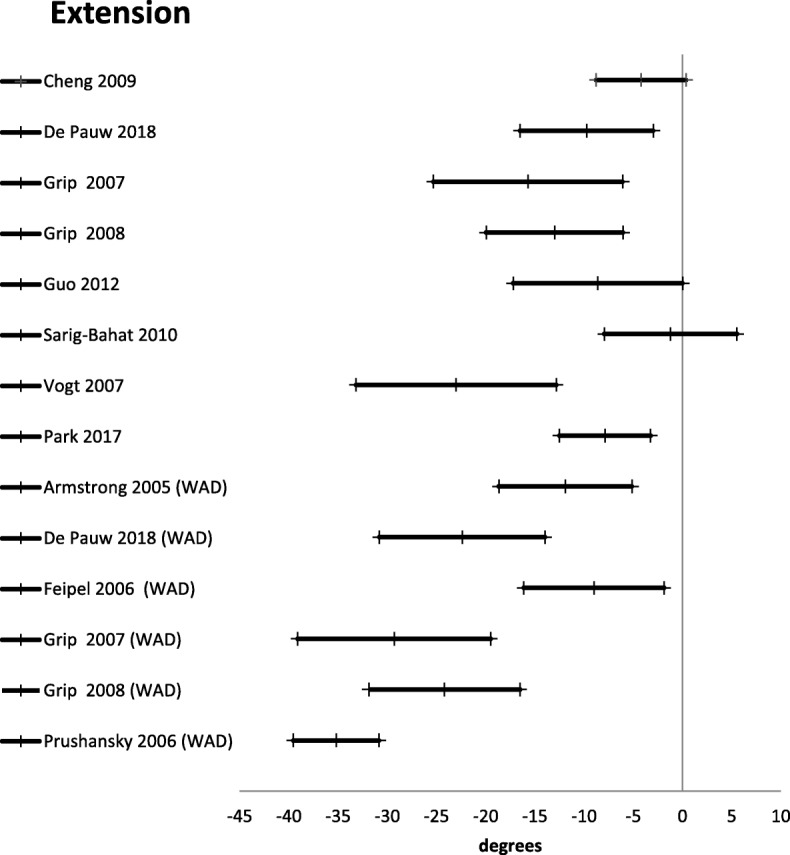
Extension. Mean difference in neck extension between people with neck pain and healthy controls

**Fig. 6:**
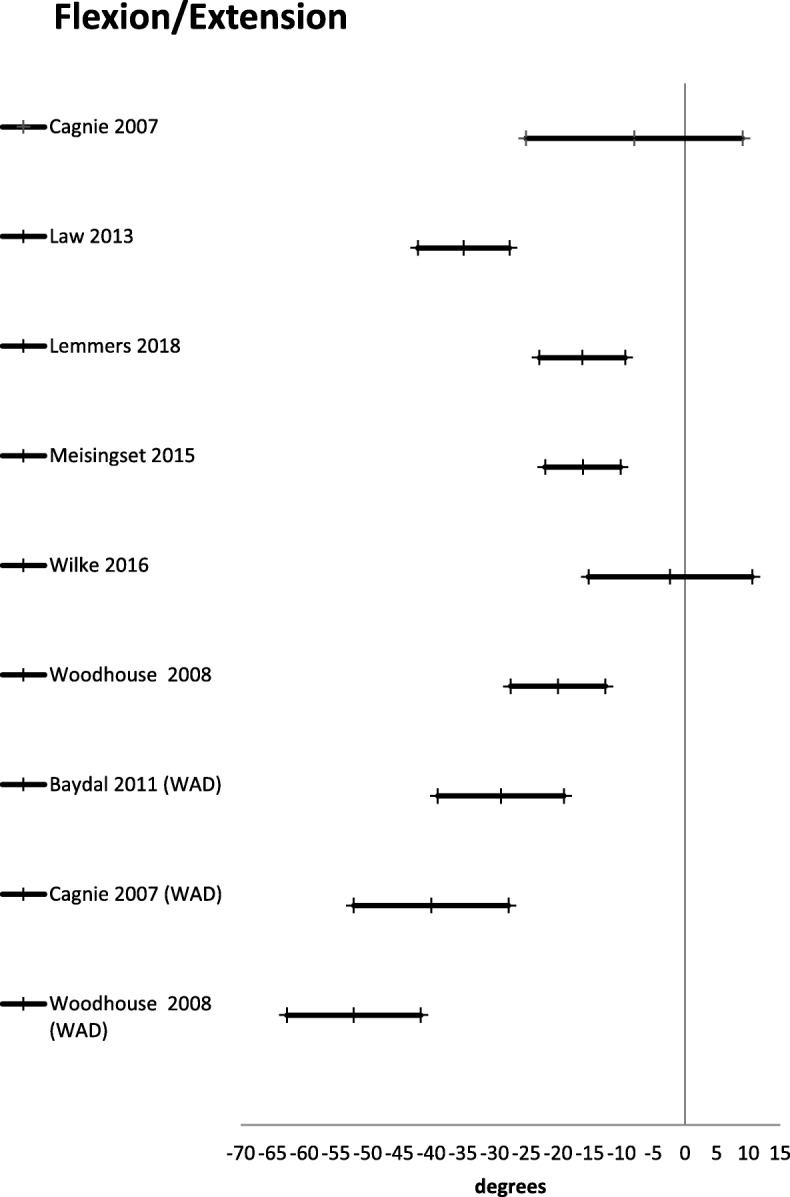
Full range flexion to extension. Mean difference in neck flexion/extension between people with neck pain and healthy controls

Active range of motion was reported in 24 studies [21, 27, 30, 42, 45, 46, 48–55, 57, 59–61, 64, 67, 69] (Table 3). There were three different ways of reporting range of motion: half cycle range of motion, which is the range from neutral starting position to end position in a given direction; full cycle range of motion, which is the range from endpoint in one direction to the opposite endpoint; and a division between upper and lower cervical range of motion in the sagittal plane. The mean difference for half cycle range of motion is presented in Figs. 2, 3, 4, 5 and 6. Across all 24 studies, people with neck pain had a smaller range of motion when compared with healthy controls.

**Table 3:** Description of studies measuring active range of neck motion

First author, year, design	Study population		Testing circumstances		Device	Comparison of range of motion		
	Type of neck pain, pain duration, pain intensity, sex (♂,♀), mean age (SD), mean BMI (SD), recruitment, occupation	Controls’ sex (♂,♀), mean age (SD), mean BMI (SD), recruitment, occupation	Examiners professional background, training, blinding	Instructions standardised, type of test, training, repeated, restrictions applied*	Type, sample rate (Hz), measurement error, LOA, ICC, SEM	Neck pain group I, type, degrees (SD)	Neck pain group II, type, degrees (SD)	Healthy controls, type, degrees (SD)
Armstrong 2005, case control	WAD II and III, 28.5 (19.5) months, NPRS 4.7 (1.6), 8♂, 15♀, 41.2 (11.9), 24.7 (4.7), local newspaper, NR	10♂, 13♀, 33.9 (12.1), 23.4 (3.2), local newspaper, NR	NR, NR, NR	Yes, AROM and JPE, NR, NR, sitting, back support, no, to pain limit, NR, blindfolded	3-Space Fastrak, 40 Hz, 0,2, NR, > 0,96, NR	WAD. F: 34.6 (8.8), E: 48.2 (13.4), LR: 61.0 (9.0), RR: 60.5 (9.3), LLF: 33.9 (7.5), RLF: 33.2 (7.0)		HC. F: 37.5 (7.5), E: 60.1(9.7), LR: 69.1 (8.8), RR: 67.4 (8.2), LLF: 37.2 (7.5), RLF: 37.3 (6.8)
Baydal 2011, case control	WAD II and III, > 6 month, < 12 months, NR (NR), 15♂, 15♀, (NR), NR (NR), rehabilitation unit, NR. Simulators, NR (NR), non-symptomatic (NR), 15♂, 14♀, NR (NR), NR (NR), IBV database, NR	15♂, 15♀, NR (NR), NR (NR), NR, NR	NR, NR, NR	Yes, repetitive flexion–extension, yes, no, sitting, back support, fixed to the back support, NR, self-selected, no	Video-photogrammetry system, NR, NR, NR, NR, NR	WAD. F/E: 90 (22)	WAD simulators. F/E:55 (24)^b^	HC. F/E: 119 (17)^ac^
Cagnie 2007, case control	INP, > 6 months, NR (NR), 0♂, 14♀, 28.3 (5.4), NR (NR), local advertisement, NR. WAD II, > 6 months (NR), NR (NR), 0♂, 16♀, 27.2 (4.8), NR (NR), local advertisement, NR	48♂, 48♀, NR (NR), NR (NR), NR, NR	NR, NR, NR	NR, AROM, yes, yes, sitting with no back support, no, end range, self-selected, no	Zebris CMS 70P US-based motion analysis system, NR, NR, NR, 0.8 < intra < 0.87, 0.92 < inter < 0.94, 5.47 < intra < 7.88, 4.25 < inter < 5.78	WAD. F/E: ≈ 113 (≈ 24), R: ≈ 137 (≈ 24), LF: ≈ 81 (≈ 16)	INP. F/E: ≈ 145^c^ (≈ 32), R: ≈ 153 (≈ 20), LF: ≈ 97^c^ (≈ 16)	HC. F/E: ≈ 153^a^ (≈ 16), R: ≈ 198^ab^ (≈ 16), LF: ≈ 97 (≈ 16)
Cheng 2009, case control	CNP, 4.4 (2.2) years, NPRS 3.7 (0.8), 6♂, 6♀, 25.4 (2.1), NR (NR), NR, graduated students, teachers, or clinicians	7♂, 5♀, 24.9 (1.8), NR (NR), NR, graduated students	NR, NR, NR	NR, neck flexion/extension, yes, NR, sitting, back support, yes, not end range, self-selected, no	Electrogoniometer (CXTLA02, Crossbow, Inc., San Jose, CA, USA), NR, 0.1°, NR, NR, NR	CNP. F: 43.5 (6.5), E: 42.8 (6.5)		HC. F: 44.2 (7.5), E: 47.0 (5.4)
De Pauw 2018, case control	INP, 86.97 (84.88) months, NPRS 2 (2.08), 0♂38♀, 38.00 (1.41), 22.75 (7.77), internet, flyers and posters, NR. WAD, 86.62 (86.66), NPRS 5 (2.70), 0♂35♀, 47.00 (1.11), 22.30 (3.64), internet, flyers and posters, NR	0♂ 30♀, 30.45 (1.15), 21.83 (3.81), internet, flyers, and posters, NR	NR, NR, NR	NR, AROM, NR, yes, sitting, unclear, NR, NR, NR	Acumar digital inclinometer, model ACU360; Lafayette Instrument Co, Lafayette, IN, USA, NR, 1°, NR, NR, NR	INP, F: 55.09 (10.02), E: 64.15 (14.78), LLF: 36.76 (7.62), RLF: 35.23 (7.62)	WAD, F: 44.64 (17.39), E: 51.50 (20.73)^c^, LLF: 33.06 (12.59), RLF: 31.94 (12.50)^c^	HC, F: 62.96 (8.73)^ab^, E: 73.89 (13.62)^b^, LLF: 41.52 (7.21)^b^, RLF: 40.68 (6.75)^ab^
Feipel 2006, case control	WAD, 31 (32) months, NR (NR), 11♂, 18♀, 37 (14), NR (NR), NR, NR	12♂, 14♀, 35 (11), NR (NR), NR, NR	NR, NR, NR	Yes, ROM (F/E and R) HRE, yes, yes, sitting back support, no, end range, self-selected, yes and no	3D electrogoniometer (CA 6000 Spine Motion Analyzer, O.S.I., Union City, CA),100 Hz, NR, NR, NR, NR	WAD. F: 54 (11), E: 47 (14)		HC. F: 64^a^ (9), E: 56 (13)
Grip 2007, case control	CNP, > 3 months, VAS 49.2(20.8) mm, 7♂, 14♀, 49 (16), NR (NR), rehabilitation clinics and medical centres, NR. WAD I and II, > 3 months, VAS 66.1 (18.8) mm, 5♂, 17♀, 49 (15), NR (NR), rehabilitation clinics and medical centres, NR	8♂, 16♀, 50 (18), NR (NR), advertisement, NR	Research assistant, NR, NR	Yes, AROM, JPE, yes, yes, sitting, NR, no, end range self-selected, eyes closed	Myrin device and ProReflex system (Qualisys Medical AB®, Gothenburg, Sweden), 120, 0.8 (1.73), NR, NR, NR	CNP. F: 52.0 (17.2), E: 43.6 (18.4), LR: 55.1 (14), RR: 54.6 (14.8)	WAD. F: 38.0 (18.4)^c^, E: 30.0 (19.4)^c^, LR: 43.1 (15.3) ^c^, RR: 44.1 (12.7)	HC. F: 61.4 (12.9)^b^, E: 59.3 (13.8)^ab^, LR: 66.8 (9.2)^ab^, RR: 67.8 (9.1)
Grip 2008, case control	CNP, > 3 months, VAS 49.2 (NR) mm, 7♂, 14♀, 49 (16), NR (NR), rehabilitation clinics and medical centres, NR. WAD I and II, > 3 months, 66.1 (18.8) mm, 5♂, 17♀, 49 (15), NR (NR), rehabilitation clinics and medical centres, NR	8♂, 16♀, 50 (18), NR (NR), advertisement, NR	NR, NR, NR	Yes, fast head rotation, yes, yes, sitting, NR, NR, pain limit, fast as possible, no	ProReflex system (Qualisys Medical AB®, Gothenburg, Sweden), 120, 0.8 (1.73), NR, NR, NR	CNP. F: 46.6 (18.0), E: 38.2 (12.3), pooled rotation: 55.7 (16.1)	WAD. F: 31.6 (18.1)^c^, E: 27.0 (14.8)^c^, pooled rotation: 41.9 (19)^c^	HC. F: 65.3 (10.8)^ab^, E: 51.2 s (11.4)^b^, pooled rotation: 71.3 (11.3)^ab^
Guo 2012, case control	NP, NR (NR), VAS 27 (20) mm, 13♂, 14♀, 24.2 (5.9), NR (NR), NR, NR	Total 13, NR♂, NR♀, 20.9 (1.3), NR (NR), NR, NR	NR, NR, NR	NR, AROM, NR, NR, sitting, NR, NR, end range, self-selected, NR	Fastrak, Polhemius, USA, 120, NR, NR, AROM: > 0, 791 conjuct motion: > 0.4, NR	NP. F: 59.7 (13.7), E: 70.8 (15.4), LR: 68.1 (9.3), RR: 63.1 (9.5), LLF: 44.4 (10.3), RLF: 46.0 (9.0)		HC. F: 62.2 (11.1), E: 79.4 (11.7)^a^, LR: 69.8 (7.1), RR: 71.2 (6.4)^a^, LLF: 46.6 (6.0), RLF: 48.6 (6.9)
Law 2013, case control	NP, NR (NR), NR (NR), 9♂, 17♀, 44.52 (7.11), NR (NR), physiotherapy department, NR	9♂, 17♀, 45.28 (9.12), NR (NR), physiotherapy department, NR	Physiotherapists, yes	Yes, AROM, yes, yes, sitting (F, E, LF), prone (R), yes, end range, slow, no	Electronic CROM goniometer, on activation, NR, NR, 0.71 < 091, 3.50 (154.72–168.44) < 6.05 (112.11–135.81)	NP. F/E: 89.09 (14.38), R: 134.42 (18.91), LF: 69.04 (12.54)		HC. F/E: 123.96 (15.12)^a^, R: 161.58 (9.36)^a^, LF: 89.19) (13.10)^a^
Lemmers 2018, case control	Non-specific, NR, 4 (2) NPRS, 16♂19♀, 48 (15), NR, physiotherapy clinic, NR	50♂ 50♀, 44 (16), NR, physiotherapy clinic, NR	NR, NR, NR	Yes, AROM, yes, yes, sitting with back support, no, pain limit, self-selected, no	‘Flock of Birds electromagnetic tracking system (Ascension Technologies, Shelburne, USA©), no, NR, NR, NR, NR’	Non-specific NP, F/E: 94.30 (17.41), R LF: 53.21 (14.44)		HC, F/E: 100.48 (18.30), LF: 57.35 (14.81)
Meisingset 2015, case control	NP, > 2 weeks, NPRS 4.6 (1.4), 20♂, 55♀, 43.1 (12.9), 24.9 (4.7), private physiotherapy clinic and specialised neck and back pain clinic at university hospital, NR	43♂, 48♀, 40.8 (13.8), 25 (3.5), university hospital, NR	Physiotherapist, well trained, NR	Unclear, AROM, JPE, unclear, yes, sitting, back support, fixed to back support, end range, self- selected, yes (JPE test)	Electromagnetic motion tracker system (Polhemus, Inc, Colchester, Vermont, USA), 240 Hz, NR, NR, NR, NR	NP (adjusted for age and gender). F/E: 110.1 (19.4), R: 128.2 (17.7), LF: 68.1 (15.5)		HC (adjusted for age and gender). F/E: 126.2^a^ (19.5), R: 140.7^a^ (17), LF: 72.6^a^ (15.1)
Park 2017, case control	UPNP, > 6 weeks, VAS 60.45 (7.70) mm, 10♂10♀, 23.45 (1.9), NR, students at Yonsei University, NR	10♂ 10♀, 23.35 (2.08), NR, students at Yonsei University, NR	NR, NR, NR	No, AROM, NR, NR, sitting with back support, yes, pain limit, NR, NR	Students at Yonsei University, Germany), 20, 1°, NR, 0.80-0.90, NR	UPNP, F: 51.60 7.49), E: 64.71 (7.10), R non-pain side: 55.31 (9.68), R pain side: 50.79 (10.01), LF non-paid side: 40.45 (4.52), LF pain side: 31.88 6.11)		HC, F: 59.73 (7.28)a, E: 72.59 (7.84)a, R non-pain side: 56.63 (7.83)a, R pain side: 58.43 (8.55), LF non-pain side: 44.92 (4.15)LF pain side: 40.11 (5.74)
Prushansky 2006, case control	WAD II and III, > 6 months < 132 months, NR (NR), 47♂, 54♀, 40.3 (10.6), (NR), NR, NR	16♂, 59♀, 36.3 (8.9) (8.9), NR (NR), NR, NR	NR, NR, NR	Yes, AROM, NR, yes, NR, NR, pain limit, self-selected, NR	Zebris CMS 70P system (Zebris Medizintechnik Gmbh, Isny, Germany), NR, NR, NR, NR, NR	WAD. F: 30.5 (15.1), E: 28.9 (13.3), LR: 43.1 (16.6), RR: 41.3 (14.3), LLF: 27.1 (9.6), RLF: 25.9 (10.0)		HC. F: 58.7 (12.7), E: 64.1 (15.5), LR: 73.9 (10.7), RR: 71.1 (9.3), LLF: 41.9 (8.0), RLF: 43.0 (7.6)
Röijezon 2010, case control	Non-specific neck pain (sample 1), 132 (NR) months, VAS 62 (16) mm, 0♂, 16♀, 48 (7), 26.6 (4.9), local papers, NR. Non-specific neck pain (sample 2), 120 (NR) months, NPRS 5.4 (1.6), 0♂, 102♀, 51 (9), 26.7 (4.7), local papers, NR	0♂, 16♀, 45 (10), 23.8 (1.7), local papers, NR 0♂, 33♀, 47 (10), 24.9 (4.1), local papers, NR	NR, NR, NR	Yes, no, fast ROM, yes, yes, sitting, NR, NR, fast as possible, no	Electromagnetic tracking system (FASTRAK™, Polhemus Inc, USA), 60 Hz, NR, NR, peak speed: HC 0.75 (0.41–0.91), NP 0.84 (0.58–0.94). ROM: HC 0.64 (0.21–86), NP 0.86 (0.63–0.95), peak speed: HC 33 (25–52), NP 41 (31–64). HC ROM: 4.2 (3.1–6.4), NP 3.8 (2.8–5.9	NP. R: 52.7 (9.2)		HC. R: 61.5 (8.3)^a^
Rudolfsson 2012, case control	CNP, 120 (NR) months, NPRS 3.5 (2.0), 0♂, 102♀, 51 (9), 26.7 (4.7), NR, NR	0♂, 33♀, 47 a (10), 24.9 (4.1), NR, NR	NR, NR, yes	Yes, F, E and R, NR, yes, sitting with back support, fixed, end range, self-selected, eyes closed	Fastrak, Polhemus Inc, USA, 60, NR, NR, NR, NR	NP. UC F: 32.6 (6.1), UC E: 40.4 (9.2), LC F: 16.0 (5.4), LC E: 3.0 (2.8), R: 115.2 (17.0)		HC. UC F:33.9 (6.0), UC E: 50.9 (8.2), LC F: 21.1 (4.5), LC E: 5.4 (4.2), R: 136.2 (15.0)
Rudolfsson 2017, cross-sectional	Non-specific neck pain, 60 (24–124) months, NPRS 4,62 (1,8), 0♂120♀, 47,3 (11,6), 24,7 (4,2), NR, NR	0♂ 40♀ 46,9 (11,8), 23,3 (2,8), NR, NR	Medical doctor, NR, NR	Yes, AROM (F/E), natural head posture, NR, yes, sitting, back support, fix to back support, far as possible, NR, NR	FASTRAK, Polhemus Inc., Colchester, VT, USA, 40 Hz, NR, NR, NR, NR	NP: UC F: 33.7 (7.0). UC E: − 46.0 (10.6). LC F: 11.8 (6.0). LC E: − 1.8 (4.7)		HC: UC F: 36.3 (7.8)^a^. UC E: − 53.3 (9.9). LC F: 16.3 (5.3). LC E: 2.6 (5.9)^a^
Sarig-Bahat 2010, case control	CNP, 43.4 (5.3), VAS 33 (20.5) mm, 9♂, 16♀, 39.0 (12.7), NR (NR), local physiotherapy clinic, NR	11♂, 31♀, 35.3 (12.4), NR (NR), University of Haifa, NR	NR, NR, NR	Yes, AROM, no, yes, sitting, yes, end range, NR, NR	Fastrak, Polhemus, 60, NR, NR, NR, NR	CNP. F: 46.1 (16.4), E: 43.1 (15.0), LR: 59.2 (11.0), RR: 57.5 (12.5)		HC. F: 58.4 (11.5), E: 44.3 (10.9), LR: 66.7 (6.6), RR: 66.3 (7.5)
Sjølander 2008, case control	Insidious neck pain, 97 (68) months, VAS 52 (16) mm, 0♂, 9♀, 40 (9), NR (NR), rehabilitation centre, NR. WAD II and III, 76 (84), VAS 45 (19) mm, 2♂, 5♀, 45 (11), NR (NR), rehabilitation centre, NR	3♂, 13♀, 41 (9), NR (NR), local community, NR	NR, NR, NR	Yes, AROM in rotation, yes, yes, standing, NA, end range, fast as possible, NR	Electromagnetic tracking system, 60, NR, NR, NR, NR	Insidious neck pain. LR: 61.8 (10.9), RR: 57.9 (11.0)	WAD. LR: 68.5 (12.9), RR: 67.7 (12.1)	HC. LR: 71.9 (13), RR: 70.7 (11.2)
Tsang 2013, case control	CNP, 5.13 years, NPRS 38.97/100, 9♂25♀, 38.44 (10.87), 21.77 (3.03), local community, NR	9♂ 25♀, 34.35 (9.08), 22.07, local community, NR	NR, NR, NR	Yes, AROM, yes, yes, sitting, NR, no, end range, NR, no	Fastrak, Polhemus Inc., Colchester, VT, USA, 30, NR, NR, NR, NR	CNP. F: ≈ 33 (4), E: ≈ 28 (6), LR: ≈ 57 (8), RR: ≈ 58 (10), LLF: ≈ 25 (8), RLF: ≈ 28 (8)		HC. F: ≈ 32 (6), E: ≈ 28 (6), LR: ≈ 61 (10), RR: ≈ 61 (8), LLF: ≈ 25 (6), RLF: ≈ 28 (69)
Vogt 2007, case control	CNP, NR (NR), WAS 37 (8) mm, 6♂, 10♀, 55.8 (2.8), NR (NR), co-operating rehabilitation clinics, NR	8♂, 10♀, 56.6 (3.5) (3.5), NR (NR), advertisements, NR	NR, NR, NR	Yes, AROM, yes, yes, sitting, NR, end range, self-selected, NR	Zebris CMS 70©, Germany, 30, > 0.6 mm, NR, > 0.92 (*r* = 0.996–0.921; *p* < 0.001), NR	CNP. F: 40.7 (19.9), E: 44.3 (18.4), LR: 52.6 (17.3), RR: 56.6 (18.9), LLF: 25.4 (9.6), RLF: 27.8 (14.0)		HC. F: 56.3 (8.5), E: 67.3 (10.2), LR: 75.3 (7.5), RR: 68.6 (6.5), LLF: 35.0 (6.6), RLF:35.1 (6.8)
Waeyaert 2016, case control	CNP,> 3 months, NR (NR), 9♂, 28♀, 45.72 (13.19), NR (NR), NR, NR	91♂, 59♀, 40.85 (13.19), NR (NR), NR, NR	Manual therapists, 2–20 years, yes	Unclear, AROM, NR, yes, sitting, NR, NR, withoutcausing discomfort, NR, NR	Electromagnetic tracking system (Flock of Birds; Ascension Technologies, Shelburne, VT), NR, NR, NR, NR, NR	CNP. F/E: NR (NR), R: NR (NR), LF: 59.28 (15.41)		HC. F/E: NR (NR), R: NR (NR), LF: 68.67^a^ (15.17)
Wilke 2016, case control	Myofascial neck pain, NR (NR), NR (NR), 9♂, 13♀, 33.4 (13.9), 22.9 (3.5), clinic for orthopaedic surgery	9♂, 13♀, 34.5 (13), 23.8 (3.6), NR, NR	NR, NR, NR	Unclear, AROM, yes, yes, sitting, NR, NR, NR, NR, NR	Three-dimensional, ultrasonic movement analysis system (Zebris CMS 70, Zebris Meditechnic GmbH, Isny, Germany), 30 Hz, > 0.6 mm, NR, 0.86-0.95, NR	CNP. F/E: 125.9 (23.2)		HC. F/E: 128.2 (20.4)
Woodhouse 2008, case control	WAD I and II, > 6 months, NPRS 5.60 (2.49), 22♂, 34♀, 38.19 (10.8), NR (NR), referred, NR. CNP, > 6 months (NR), NPRS 3.84 (− 1.74), 19♂, 38♀, 43.7 (12.6), NR (NR), physiotherapists and general practitioners, NR	29♂, 28♀, 38.2 (10.9), NR (NR), different, NR	NR, NR, No	NR, AROM, NR, yes, sitting, NR, end range, NR, eyes closed	3-Space Fastrak, 120 Hz, reference, NR, NR, NR	WAD. F/E: 81.8 (34.6), R: 106.2 (34.7), LF: 60.9 (18.8)	CNP. F/E: 114.0 (20.0)^c^, R: 133.1 (18.6)^c^, LF: 72.2 (13.2)^c^	HC. F/E: 134.0 (20.7)^ab^, R: 151.7 (16.5)^ab^, LF: 84.9 (13.8)^ab^

*Abbreviations*: *AROM* active range of motion, *BMI* body mass index, *CNP* chronic neck pain, *F/E* flexion/extension, *HC* healthy controls, *HRE* head repositioning error, *ICC* intraclass correlation coefficients, *INP* idiopathic neck pain, *JPE* joint positioning error, *LF* lateral flexion, *LLF* left lateral flexion, *LOA* limits of agreement, *LR* left rotation, *NP* neck pain, *NPRS* numeric pain rating scale, *NR* not reported, *R* rotation, *RLF* right lateral flexion, *RR* right rotation, *UPNP* unilateral posterior neck pain, *SD* standard deviation, *SEM* standard error of measurement, *VAS* visual analog scale, *WAD* whiplash-associated disorder

*Is test position in sitting, standing, with back support? Is the test subject fixed to the back support? Test to end range or pain limit? Movement speed self-selected, fast as possible or fixed? Is the test subject blindfolded?

^a^Significant difference neck pain group I vs HC (*p* > 0,05)

^b^Significant difference neck pain group II vs HC (*p* > 0,05)

^c^Significant difference neck pain group I vs II (*p* > 0,05). ≈ Red from a graph

### Quality of motion

Quality of motion was addressed in 15 studies [27, 29, 30, 44, 45, 48–51, 54, 57, 59, 63, 64] (Table 4). Cervical movement speed was reported as peak velocity in seven studies [44, 45, 48, 54, 57, 63, 64] and average movement velocity in six [29, 30, 44, 50, 57, 63]. In five studies [44, 45, 50, 63, 64], significantly lower movement speed was reported for the neck pain groups compared with the healthy controls whereas in the remaining studies, the differences between groups were not statistically significant. Of the six studies on conjunct motion [27, 48, 51, 54, 64], two studies [27, 64] reported less conjunct motion for the neck pain groups compared with the healthy controls. In the remaining four studies, no differences between the groups were found.

**Table 4:** Description of the studies measuring quality of neck motion

First author, year, design	Study population		Testing circumstances		Device	Comparison of motion quality		
	Type of neck pain, pain duration, pain intensity, sex (♂,♀), mean age (SD), mean BMI (SD), recruitment, occupation	Controls’ sex (♂,♀), mean age (SD), mean BMI (SD), recruitment, occupation	Examiners professional background, training, blinding	Instructions standardised, type of test, training, repeated, restrictions applied*	Type, sample rate (Hz), measurement error, LOA, ICC, SEM	Neck pain group I, type of measurement, degree (SD)	Neck pain group II, type of measurement, degree (SD)	Healthy controls, type of measurement, degree (SD)
Alsultan 20.19, cross sectional	CNP and WAD, > 3 month, NPRS 4.08 (1.89), 8**♂**10**♀**, 32.22 (13.41), NR, NR, NR	8**♂** 10**♀**, 25.89 (7.34), NR, NR, NR	NR, NR, NR	Yes, repeated movement within mid-range and three speeds, yes, yes, sitting with back support, no, within mid-range, slow, natural and fast, no	Optoelectronic system (BTS Bioengineering, Milan, Italy), 250, NR, NR, NR, NR	CNP. Helical axis (mean distance): F/E slow: 1.39 cm (0.25 cm), F/E natural: 1.46 cm (0.33 cm), F//E fast: 1.65 cm (0.39 cm), LF slow: 0.90 cm (0.23 cm), LF natural: 0.91 cm (0.23 cm), LF fast: 0.91 cm (0.25 cm), R slow: 0.90 cm (0.29 cm), R natural: 0.83 cm (0.15 cm), R fast: 0.84 cm (0.15 cm). Helical axis (mean angle): F/E slow: 4.22° (0.57°), F/E natural: 4.51° (0.73°), F//E fast: 3.88° (0.75°), LF slow: 8.61° (1.92°), LF natural: 8.96° (1.62°), LF fast: 9.04° (2.07°), R slow: 4.89° (0.71°), R natural: 4.98° (0.85°), R fast: 3.98° (0.42°).		HC. Helical axis (mean distance): F/E slow: 1.63 cm (0.31 cm)^a^, F/E natural: 1.61 cm (0.28 cm)^a^, F//E fast: 1.71 cm (0.31 cm)^a^, LF slow: 0.93 cm (0.34 cm), LF natural: 1.02 cm (0.44 cm), LF fast: 0.97 cm (0.31 cm), R slow: 0.93 cm (0.22 cm)^a^, R natural: 1.07 cm (0.33 cm)a, R fast: 0.99 cm (0.35 cm)a. Helical axis (mean angle): F/E slow: 4.39° (0.99°), F/E natural: 4.29° (0.91°), F//E fast: 3.89° (0.92°), LF slow: 9.21° (2.42°), LF natural: 9.70° (2.16°), LF fast: 9.20° (2.11°), R slow: 5.44° (1.64°)^a^, R natural: 5.21° (1.04°)a, R fast: 4.99° (1.02°)^a^
Bahat 2010, case control	CNP, 43.4 (5.3) months, VAS 3.3 (2.05), 9♂16♀, 39.0 (12.7), NR, local physiotherapy clinic, NR	11♂, 31♀, 35.3 (12.4), NR (NR), University of Haifa, NR	physiotherapist, experienced, NA	Yes, fast movement test, yes, yes, sitting, yes, to a target, fast as possible, no	Fastrak: Polhemus, 40 Hercules, 60 Hz, NR, NR, NR, NR	CNP. Mean velocity (°/sec). F: 24.4 (9.1). E: 29.1 (12.4). LR: 36.9 (15.2). RR: 39.8 (16.2). Peak velocity (°/sec^2^). F: 69.8 (34.7). E: 81.4 (39.7). LR: 108.5 (48.3). RR: 100.2 (43.6). Number of velocity peaks: F: 6.1 (2.6). E: 5.9 (2.1). LR: 6.1 (3.3). RR: 4.9 (2.8)		HC. Mean velocity (°/sec) F: 33.3 (12.6). E: 46.5 (16.3). LR: 56.4 (13.6). RR: 55.0 (15.9). Peak velocity (°/sec^2^) F: 105.0 (43.0). E: 138.6 (52.7). LR: 165.9 (51.9). RR: 162.2 (56.6). Number of velocity peaks: F: 5.0 (1.7). E: 4.8 (1.8). LR: 4.2 (1.8). RR: 3.6 (1.4)
Bahat 2015, case control	CNP, 93.03(104.46) months, VAS 36.42 (17.24) mm, 13♂, 20♀, 37.56 (9.95), NR (NR), University of Queensland and University of Haifa	15♂, 8♀, 33 (678), NR (NR), University of Queensland and University of Haifa, NR	Physiotherapists, experienced, NR	Yes, hit a target, yes, yes, sitting, fixed to back support, 40° of range, fast as possible, no	Head mounted display with a built-in motion tracker (Wrap™ 1200VR by Vuzix, New York, www.vuzix.com), 30, NR, NR, NR, NR	CNP. Mean velocity (°/sec): F: 20.14 (9.0), E: 29.08 (17.1), LR: 34.91 (14.0), RR: 32.88 (15.2). Peak velocity (°/sec): F: 50.34 (20.2), E: 55.30 (30.3), LR: 73.73 (28.7), RR: 66.64 (31.1). Number of velocity peaks: F: 1.24 (0.8), E: 1.40 (1.2), LR: 2.23 (1.0), RR: 1.99 (1.2)		HC. Mean velocity (°/sec): F: 83.82^a^ (33.3), E: 76.89^a^ (33.1), LR: 100.60^a^ (3.5), RR: 100.69^a^ (40.1). Peak velocity (°/sec^2^): F: 166.82^a^ (69.6), E: 149.05^a^ (68.6), LR: 261.59^a^ (104.4), RR: 220.93^a^ (89.2). Number of velocity peaks: F: 2.79^a^ (1.1), E: 2.42^a^ (1.3), LR: 3.5^a^ (1.2), RR: 3.5^a^ (1.3)
Baydal 2011, case control	WAD II and III, > 6 month, < 12 months, NR (NR), 15♂, 15♀, (NR), NR (NR) rehabilitation unit, NR. simulators, NR (NR), non-symptomatic WAD, (NR), 15♂, 14♀, NR (NR), NR (NR), IBV database, NR	15♂, 15♀, NR (NR), NR (NR), NR, NR	NR, NR, NR	Yes, repetitive flexion–extension, yes, no, sitting, back support, fixed to the back support, NR, self-selected, no	Video-photogrammetry system, NR, NR, NR, NR, NR	WAD II and III. Peak velocity (°/sec). F/E: 71 (22) WAD II and III. Peak acceleration (°/sec^3^). F/E: 168 (93)	WAD simulators. Peak velocity (°/sec). F/E: 29 (16) WAD simulators. Peak acceleration (°/sec^3^). F/E: 59 (36)	HC. Peak velocity (°/sec). F/E: 149 (50)^ab^, HC. Peak acceleration (°/sec^3^). F/E: 410 (200)^ab^
Cheng 2009, case control	CNP, 4.4 (2.2 ) years, NPRS 3.7 (0.8), 6♂, 6♀, 25.4 (2.1), NR (NR), NR, graduated students, teachers, or clinicians	7♂, 5♀, 24.9 (1.8), NR (NR), NR, graduated students	NR, NR, NR	NR, neck flexion/extension, yes, NR, sitting, back support, yes, not end range, self-selected, no	Electrogoniometer (CXTLA02, Crossbow, Inc., San Jose, CA, USA), NR, 0.1°, NR, NR, NR	CNP. Mean velocity (°/sec). F: 9.0 (2.1). E: 9.2 (1.3)		HC. Mean velocity (°/sec). F: 9.8 (3.8). E: 10.7 (2.7)
Feipel 2006, case control	WAD, 31 (32) months, NR (NR), 11♂, 18♀, 37 (14), NR (NR), NR, NR	12♂, 14♀, 35 (11), NR (NR), NR, NR	NR, NR, NR	Yes, AROM (F/E and R ) HRE, yes, yes, sitting back support, no, end range , self-selected, yes and no	3D electrogoniometer (CA 6000 Spine Motion Analyzer, O.S.I., Union City, CA),100 Hz, NR, NR, NR, NR	WAD. Peak velocity (°/sec). F: 121 (48. E: 118 (44). F-conjuct R: 2 (4), F-conjuct LF: 2 (5), E-conjuct R: 5 (6), E-conjuct LF: 1 (4)		HC. Peak velocity (°/sec). F: 157 (85). E: 147 (77). F-conjuct R: 2 (5), F-conjuct LF: 1 (5), E-conjuct R: 3 (6), E-conjuct LF: 4 (4)^a^
Grip 2008, case control	CNP, > 3 months, VAS 49.2 (NR) mm, 7♂, 14♀, 49 (16), NR (NR), rehabilitation clinics and medical centres, NR. WAD I and II, > 3 months, VAS 66.1 (18.8) mm, 5♂, 17♀, 49 (15), NR (NR), rehabilitation clinics and medical centres, NR	8♂, 16♀, 50 (18), NR (NR), advertisement, NR	NR, NR, NR	Yes, fast head rotation, yes, yes, sitting, NR, NR, pain limit, fast as possible, no	ProReflex system (Qualisys Medical AB®, Gothenburg, Sweden), 120, 0.8 (1.73), NR, NR, NR	CNP. Mean velocity (°/sec). F: 44.6 (29.8). E: 60.2 (39.0). Pooled rotation: 60.2 (39.0)	WAD. Mean velocity (°/sec). F: 28.4 (22.7)^c^. E: 21.1 (15.9)^c^. Pooled rotation: 43.3 (30.4)^c^	HC. Mean velocity (°/sec). F: 72.3 (21.7)^ab^. E: 59.3 (20.7)^ab^. Pooled rotation: 95.4 (29.9)^ab^
Guo 2012, case control	NP, NR (NR), 27 (20) mm, 13♂, 14♀, 24.2 (5.9), NR (NR), NR, NR	Total 13, NR♂, NR♀, 20.9 (1.3), NR (NR), NR, NR	NR, NR, NR	NR, AROM, NR, NR, sitting, NR, NR, end range, self-selected, NR	Fastrak, Polhemius, USA, 120, NR, NR, AROM: > 0,791 Conjuct motion: > 0.4, NR	NP. F-conjuct RLF: 5.8 (3.3), F-conjuct RR: 6.3 (3.4), E-conjuct LLF: 8.3 (4.7), E-conjuct LR: 6.6 (2.8), LR-conjuct E: 9.9 (7.4), LR-conjuct LLF: 11.7 (7.9), RR-conjuct E: 8.8 (8.7), RR-conjuct RLF: 8.6 (9.1), LLF-conjuct F: 11.3 (7.1), LLF-conjuct F: 11.3 (7.1), LLF-conjuct LR: 16.7 (8.2), RLF-conjuct F: 13.9 (6.4), RLF-conjuct RR: 18.5 (7.2)		HC. F-conjuct RLF: 5.0 (2.5), F-conjuct RR: 4.5 (1.6)^a^, E-conjuct LLF: 7.9 (4.7), E-conjuct LR: 7.3 (2.9), LR-conjuct E: 8.4 (3.8), LR-conjuct LLF: 12.0 (3.8), RR-conjuct E: 9.6 (2.9), RR-conjuct RLF: 10.7 (5.0), LLF-conjuct F: 10.2 (5.2), LLF-conjuct F: 10.2 (5.2), LLF-conjuct LR: 16.6 (7.3), RLF-conjuct F: 10.2 (5.6), RLF-conjuct RR: 16.6 (6.0)
Lemmers 2018, case control	Non-specific, NR, NPRS 4 (2), 16♂19♀, 48 (15), NR, physiotherapy clinic, NR	50♂ 50♀, 44 (16), NR, physiotherapy clinic, NR	NR, NR, NR	Yes, AROM, yes, yes, Sitting with back support, no, pain limit, self-selected, no	Flock of Birds electromagnetic tracking system (Ascension Technologies, Shelburne, USA©), no, NR, NR, NR, NR	Non-specific NP. Variability. F/E: 0.73 (0.32). LF: 0.45 (0.27). Non-specific NP. Root mean square of jerk. F/E: 12.97 (5.01). LF: 8.70 (3.62). Conjuct motion: F/E conjuct R: 18.84 (11.22), F/E conjuct LF: 15.12 (8.02), LF conjuct F/E: 59.25 (37.94), LF conjuct R: 59.01 (31.08)		HC. Variability. F/E: 0.98 (0.59). LF: 0.48 (0.31). HC. Root mean square of jerk. F/E: 13.26 (5.48). LF: 8,18 (3.67). Conjuct motion: F/E conjuct R: 18.84 (11.22), F/E conjuct LF: 15.12 (8.02), LF conjuct F/E: 59.25 (37.94), LF conjuct R: 59.01 (31.08)
Meisingset 2015, case control	NP, > 2 weeks, NPRS 4.6 (1.4), 20♂, 55♀, 43.1 (12.9), 24.9 (4.7), private physiotherapy clinic and specialised neck and back pain clinic at university hospital, NR	43♂, 48♀, 40.8 (13.8), 25 (3.5), university hospital, NR	Physiotherapist, well trained, NR	Unclear, AROM, JPE, unclear, yes, sitting, back support, fixed to back support, end range, self-selected, yes (JPE test)	Electromagnetic motion tracker system (Polhemus, Inc, Colchester, Vermont, USA), 240 Hz, NR, NR, NR, NR	NP. Peak velocity (°/sec) (adjusted for age and gender): F/E: 70.6 (35.8), R: 109.3 (45), LF: 57.9 (24.8). Conjuct motion (°) (adjusted for age and gender). F/E cunjuct: 12.3 (4.4), R cunjuct: 19.8 (8), LF cunjuct: 45.7 (24.7)		NP. Peak velocity (°/sec) (adjusted for age and gender): F/E: 115.6^a^ (35), R: 158.9^a^ (45.8), LF: 85.7^a^ (24.3). Conjuct motion (°) (adjusted for age and gender). F/E cunjuct: 16.5^a^ (4.4), R cunjuct: 25.1^a^ (7.8), LF cunjuct: 62.5 (24.3)
Röijezon 2010, case control	Non-specific neck pain (sample 1), 132 (NR) months, VAS 62 (16) mm, 0♂, 16♀, 48 (7), 26.6 (4.9), local papers, NR. Non-specific neck pain (sample 2), 120 (NR) months, NRS 5.4 (1.6), 0♂, 102♀, 51 (9), 26.7 (4.7), local papers, NR	0♂, 16♀, 45 (10), 23.8 (1.7), local papers, NR 0♂, 33♀, 47 (10), 24.9 (4.1), local papers, NR	NR, NR, NR	Yes and no, fast ROM, yes, yes, sitting, NR, NR, fast as possible, no	Electromagnetic tracking system (FASTRAK™,Polhemus Inc, USA), 60 Hz, NR, NR, peak speed: hc 0.75 (0.41–0.91), np 0.84 (0.58–0.94). ROM: hc 0.64 (0.21–86), np 0.86 (0.63–0.95), peak speed: hc 33 (25–52), np 41 (31–64). hc ROM: 4.2 (3.1–6.4), np 3.8 (2.8–5.9)	NP. Peak velocity (°/sec). R: 226 (88). SID (%): 18.1 (6.5), CM 12.4 (3.7)		HC. Peak velocity (°/sec). R: 348 (92) SID (%):13.7 (4.7) CM 18.8 (7.5)
Sjølander 2008, case control	Insidious neck pain, 97 (68) months, VAS 52 (16) mm, 0♂, 9♀, 40 (9), NR (NR), rehabilitation centre, NR. WAD II and III, 76 (84) months, VAS 45 (19) mm, 2♂, 5♀, 45 (11), NR (NR), rehabilitation centre, NR	3♂, 13♀, 41 (9), NR (NR), local community, NR	NR, NR, NR	Yes, AROM in rotation, yes, yes, standing, NA, yes, fast as possible, NR	Electromagnetic tracking system, 60, NR, NR, NR, NR	Insidious neck pain. Peak velocity (°/sec): **out-left: 95.3 (27.5), out-right: C12, in-left:89.7 (26.3), in-right: 85.4 (32.4), total jerk index: out-left: 15.9 (9.5), out-right: 14.1 (9.7), in-left: 16.2 (8.7), in-right:17.3 (11.3)	WAD. Peak velocity (°/sec): out-left: 108.6 (20.4), out-right: C12, in-left: 103.7 (19.1), in-right: 96.4 (27.4), total jerk index: out-left: 13.1 (5.1), out-right: 13.6 (9.1), in-left: 11.1 (6.1), in-right: 15.2 (10.6)	HC. Peak velocity (°/sec): out-left: 132.8 (39.6), out-right: C12, in-left: 119.5 (28.6), in-right: 116.9 (28.6), total jerk index: out-left: 7.5 (4.4)^ab^, out-right: 7.7 (4.2), in-left: 8.6 (4.9)^ab^, in-right: 9.4 (7.1)
Tsang 2013, case control	CNP, 5.13 (NR) years, NPRS 38.97/100, 9♂25♀, 38.44 (10.87), 21.77 (3.03), local community, NR	9♂ 25♀, 34.35 (9.08), 22.07, local community, NR	NR, NR, NR	Yes, AROM, yes, yes, sitting, NR, no, end range, NR, no	Fastrak, Polhemus Inc., Colchester, VT, USA, 30, NR, NR, NR, NR	CNP. Mean velocity (°/sec). F: ≈ 62 (21). E: ≈ 57 (16). LR: ≈ 134 (52). RR: ≈ 129 (52). LLF: ≈ 52 (16). RLF: ≈ 52 (10)		HC. Mean velocity (°/sec). F: ≈ 83 (26). E: ≈ 78 (26). LR: ≈ 186 (52). RR: ≈ 181 (52). LLF: ≈ 72 (31). RLF: ≈ 72 (31).
Vogt 2007, case control	CNP, NR (NR), VAS 37 (8) mm, 6♂, 10♀, 55.8 (2.8), NR (NR), co-operating rehabilitation clinics, NR	8♂, 10♀, 56.6 (3.5) (3.5), NR (NR), advertisements, NR	NR, NR, NR	Yes, AROM, yes, yes, sitting, NR, end range, self-selected, NR	Zebris CMS 70©, Germany, 30, > 0.6 mm, NR, > 0.92 (*r* = 0.996–0.921; *p* < 0.001), NR	CNP. CV (%). F: 12.1 (12.0). E: 11.2 (9.5). LR: 8.0 (7.1). RR: 8.0 (7.1). LLF: 12.0 (3.3). RLF: 13.3 (4.0).		HC. CV (%). F: 2.8 (1.3). E: 1.9 (0.9). LR: 1.7 (1.0). RR: 1.7 (1.0). LLF: 5.3 (3.0). RLF: 5.1 (2.6).
Woodhouse 2008, case control	WAD I and II, > 6 months (NR), NPRS 5.60 (2.49), 22♂, 34♀, 38.19 (10.8), NR (NR), referred, NR. CNP, > 6 months (NR), NPRS 3.84 (− 1.74), 19♂, 38♀, 43.7 (12.6), NR (0), physiotherapists and general practitioners, NR	29♂, 28♀, 38.2 (10.9), NR (NR), different, NR	NR, NR, no	NR, AROM, NR, yes, sitting, NR, end range, NR, eyes closed	3-Space Fastrak , 120 Hz, reference, NR, NR, NR	WAD. F/E-conjuct R: 3.95 (1.89), F/E-conjuct LF: 3.66 (1.82), R conjuct-F/E: 6.89 (3.22), R conjuct-LF: 7.00 (3.52), R conjuct-LF: 7.00 (3.52), LF-conjuct F/E: 6.59 (2.98)	CNP. F/E-conjuct R: 4.58 (1.32)^c^, F/E-conjuct LF: 4.18 (1.24), R-conjuct F/E: 8.99 (3.26)^c^, R-conjuct LF: 8.87 (4.09)^c^, R-conjuct LF: 8.87 (4.09)^c^, LF-conjuct F/E: 7.57 (3.12)	HC. F/E-conjuct R: 5.10 (1.53)^a^, F/E-conjuct LF: 5.24 (2.26)^ab^, R-conjuct F/E: 12.79 (5.23)^ab^, R-conjuct LF: 13.15 (4.08)^ab^, R-conjuct LF: 13.15 (4.08)^ab^, LF-conjuct F/E: 8.43 (2.69)^ab^

*Abbreviations*: *AROM* active range of motion, body mass index, *CNP* chronic neck pain, *CV* coefficient of variation, *CM* conjunct motion, *F/E* flexion/extension, *HC* healthy controls, *HRE* head repositioning error, *ICC* intraclass correlation coefficients, *JPE* joint poisoning error, *LF* lateral flexion, *LOA* limits of agreement, *LLF* left lateral flexion, *LR* left rotation, *NA* not applicable, *NPRS* numeric pain rating scale, *NP* neck pain, *NR* not reported, *R* rotation, *RLF* right lateral flexion, *RR* right rotation, *SD* standard deviation, *SEM* standard error of measurement, *SID* speed index of deviance, *VAS* visual analog scale, *WAD* whiplash-associated disorder

*Is test position in sitting, standing, with back support? Is the test subject fixed to the back support? Test to end range or pain limit? Movement speed self-selected, fast as possible or fixed? Is the test subject blindfolded?

**‘Out’ is the velocity measurement on the way from neutral to endpoint and ‘in’ is the velocity measurement from endpoint back to neutral

^a^Significant difference neck pain group I vs HC (*p* > 0,05)

^b^Significant difference neck pain group II vs HC (*p* > 0,05)

^c^Significant difference neck pain group I vs II (*p* > 0,05)

### Proprioception

Joint reposition sense was reported in 12 studies [27, 30, 34, 42, 47–49, 62–64, 66], and characteristics are described in Table 5. In eight studies, a neutral task [27, 30, 42, 47–49, 62, 64] including variables of absolute error [42, 48, 49, 62], constant error [30, 42], variable global error [30, 42], root mean square error [30], and maximal overshoot [49] was reported. In two studies, a mid-range task reporting on absolute [43, 69], constant, and variable global error was reported, and in three papers [48, 64, 65], no specification of the error measurement parameter was reported. All nine studies showed smaller joint positioning error for the healthy controls compared with the neck pain groups; the difference was statistically significant in five studies [27, 34, 47, 64]. In four studies, a task of following a motion pattern was assessed [34, 63, 64, 66], three of which [34, 63, 66] reported a significantly smaller degree of error for the healthy controls and the fourth [64] a significantly smaller degree of error for the neck patient group.

**Table 5:** Description of studies measuring joint position sense

First author, year, design	Study population		Testing circumstances		Device	Comparison of joint position sense between groups		
	Type of neck pain, pain duration, pain intensity, sex (♂,♀), mean age (SD), mean BMI (SD) recruitment, occupation	Controls’ sex (♂,♀), mean age (SD), mean BMI (SD), recruitment, occupation	Examiners professional background, training, blinding	Instruction standardised, type of test, training, repeated, restrictions applied*	Type, sample rate (Hz), measurement error, LOA, ICC, SEM	Neck pain group I, type degrees (SD)	Neck pain group II, type degrees (SD)	Healthy controls type degrees (SD)
Alahmari 2017, cross-sectional	NP, 24 (10.8) weeks, 48.6 mm (21.3), NR**♂**NR (Total 42) **♀**, 47.4 (18.8), 23.8 (3.2), physical therapy clinic, NR	NR**♂** NR total 42**♀**, 47.8 (15.2), 25.9 (3.4), advertisement, NR	NR, NR, NR	Yes, mid-range task, NR, yes, sitting (F/E task), supine (rotation task), back support, yes, NR, NR, NR	Digital inclinometer (Dualer IQ; JTECH Medical, Salt Lake City, UT), NR, NR, NR, NR, NR	NP, mid-range task: F: 6.79 (1.41), E: 7.74 (1.66), RR: 6.33 (0.79), RL: 7.21 (1.32)		HC, mid-range task: F: 2.57 (1.30)^a^, E: 2.95 (1.10)a, RR: 1.95 (1.44)a, RL: 2.14 (1.57)^a^
Armstrong 2005, case control	WAD II and III, 28.5 (19.5) months, NPRS 4.7 (1.6), 8♂, 15♀, 41.2 (11.9), 24.7 (4.7), local newspaper, NR	10♂, 13♀, 33.9 (12.1), 23.4 (3.2), local newspaper, NR	NR, NR, NR	Yes, yes, AROM and JPE, NR, NR, Sitting, back support, No, to pain limit, NR, blindfolded	3-Space Fastrak, 40 Hz, 0,2, NR, > 0,96, NR	WAD. Mid-range tasks: 2.97 (1.15), neutral range tasks: 3.55 (1.72)		HC. Mid-range tasks: 2.43 (0.62), neutral range tasks: 3.25 (2.32)
Bahat 2015, case control	CNP, 93.03 (104.46) months, VAS 36.42 (17.24) mm, 13♂, 20♀, 37.56 (9.95), NR (NR), University of Queensland and University of Haifa	15♂, 8♀, 33 (678), NR (NR), University of Queensland and University of Haifa, NR	Physiotherapists, experienced, NR	Yes, hit a target, yes, yes, sitting, fixed to back support, 40° of range, fast as possible, no	Head mounted display with a built-in motion tracker (Wrap™ 1200VR by Vuzix, New York, www.vuzix.com), 30 Hz, NR, NR, NR, NR	CNP. Follow a target: F (x,y): (25.53 (12), 70.83 (28.7), E (x,y): (36.16 (21.4), 59.76 (24.3)), LR (x,y): (57.4 (20.9), 30.62 (14.8), RR (x,y): (48.98 (22.7), 27.52 (11.2)		HC. Follow a target: F (x,y): (15.31a (4.6), 44.51^a^ (11.6), E (x,y): (22.89 (28), 34.12^a^ (8.3), LR (x,y): (43.35^a^ (16.7), 27.16 (8.1), RR (x,y): (36.00^a^ (8.4), 25.73 (7.5)
Dugailly 2015, case control	CNP, > 6 months, NR (NR), 11♂, 24♀, 42 (8), NR (NR), NR, NR.	14♂, 22♀, 42 (5), NR (NR), NR, NR	NR, NR, NR	NR, repositioning task, NR, yes, sitting, back support, no, NR, unclear, yes	Three dimensional electrogoniometer (OSI CA 6000 Spine Motion Analyzer), NR, NR, NR, NR, NR	CNP. slow RR: 4.9 (2.5), slow LR: 5.2 (2.7), slow F: 4.8 (2.6), slow E: 6.8 (3.3), fast RR: 5 (2.3), fast LR: 5 (3), fast F: 5.2 (2.8), fast E: 7.1 (3.6)		HC. slow RR: 3.1a (1.3), slow LR: 2.8^a^ (1.3), slow F: 2.6a (1.5), slow E: 3.1a (1.4), fast RR: 2.8a (1.7), fast LR: 2.9^a^ (1.2), fast F: 3.2^a^ (2.7), fast E: 3.6^a^ (2.1)
Cheng 2009, case control	CNP, 4.4 (2.2 ) years, NPRS 3.7 (0.8), 6♂, 6♀, 25.4 (2.1), NR (NR), NR, graduated students, teachers, or clinicians	7♂, 5♀, 24.9 (1.8), NR (NR), NR, graduated students	NR, NR, NR	NR, neck flexion/ extension, yes, NR, sitting, back support, yes, not end range, self-selected, no	Electrogoniometer (CXTLA02, Crossbow, Inc., San Jose, CA, USA), NR, 0.1°, NR, NR, NR	CNP, neutral flexion neutral (CE): 7.1 (3.5), neutral extension neutral (CE): 6.3 (4.7)		HC, neutral flexion neutral (CE): 3.5^a^ (1.8), neutral extension neutral (CE): 4.2^a^ (3.3)
Edmondston 2007, case control	Postural neck pain, 5.2 (4.28) years, VAS 48.3 (14.81) mm, 10♂11♀, 29.0 (7.36), NR, advertisement, NR	10♂ 12♀, 25.7 (5.95), NR, advertising, NR	Experienced physiotherapist, NR, NR	Yes, habitual sitting posture, perceived good posture and JPE, yes, yes, sitting, no, NA, NA, blindfolded	System (PEAK Performance Technologies Inc., Centen-PEAK Performance Technologies Inc., Centennial, CO, USA), 50 Hz,5 mm, NR, NR, NR	Postural neck pain, cervicothoracic angle: ≈ 2.1 (1.25), head tilt angle: ≈ 3.3 (2,6), Cx protraction angle: ≈ 2.5 (1.9), shoulder protraction angle: ≈ 1.4 (1,0)		HC, cervicothoracic angle: ≈ 1.4 (0.75), head tilt angle: ≈ 2,9 (2.1), Cx protraction angle: ≈ 2.7 (1.7), shoulder protraction angle: ≈ 1.3 (0.8)
Feipel 2006, case control	WAD, 31 (32) moths, NR (NR), 11♂, 18♀, 37 (14), NR (NR), NR, NR	12♂, 14♀, 35 (11), NR (NR), NR, NR	NR, NR, NR	YES, ROM (F/E and R) HRE, yes, yes, sitting back support, no, end range, self-selected, yes and no	3D electrogoniometer (CA 6000 Spine Motion Analyzer, O.S.I., Union City, CA),100 Hz, NR, NR, NR, NR	WAD, neutral F/E: 3.5 (2.4), neutral R: 1.1 (1.1), neutral LF: 0.8 (0.6)		HC, neutral F/E: 2.1 (2.0), neutral R: 0.6 (0.5), neutral LF: 0.4 (0.3)
Grip 2007, case control	CNP, > 3 months, VAS 49.2 (20.8) mm, 7♂, 14♀, 49 (16), NR (NR), rehabilitation clinics and medical centres, NR. WAD I and I, > 3 months, VAS 66.1 (18.8) mm, 5♂, 17♀, 49 (15), NR (NR), rehabilitation clinics and medical centres, NR	8♂, 16♀, 50 (18), NR (NR), advertisement, NR	Research assistant, NR, NR	Yes, AROM, JPE, yes, yes, sitting, NR, no, end range self-selected, eyes closed	Myrin device and ProReflex system (Qualisys Medical AB, Gothenburg, Sweden), 120, 0.8 (1.73), NR, NR, NR	CNP, absolute error F: 2.8 (1.2), E: 2.9 (1.3), RR: 3.7 (1.6), LR: 3.6 (3.0)	WAD, absolute error F: 3.4 (1.6), E: 3.5 (1.8), RR: 3.7 (1.9), LR: 4.0 (2.1)	HC, absolute error F: 2.9 (0.9), E: 2.7 (1.0), RR: 3.1 (1.3), LR: 3.5 (1.3)
Harvie 2016, case control	CNP, 12(10) years, NDI 29% (0.13), 6♂, 18♀, 44 (15), NR (NR), local physical therapy clinics, NR	6♂, 18♀, 45 (15), NR (NR), local physical therapy, BodyinMind.org website, university campus noticeboards, NR	NR, NR, NR	Yes, relocation to neutral, NR, yes, sitting, back support, fixed to back support, set limit, NR, no	Oculus VR, Menlo Park, California, NR, NR, NR, NR, NR	CNP. Absolute error: 3.3 (1.5)		HC. Absolute error: 2.8 (1.1)
Kristjansson 2004, case control	WAD II and III, > 6 months, < 6 years, NR, 0♂20♀, NR, BR, physiotherapy clinics, NR	0♂ 20♀, NR, NR, NR, NR	NR, NR, yes	Yes, motion trekking ‘the fly’, yes, yes, sitting, back support, no, NA, fixed, no	3-Space Fastrak system, NR, 0.2°, > 0.6, < 0.86, NR, NR	WAD, movement pattern A: 5.17, movement pattern B: 4.65, movement pattern C: 4.97		HC, movement pattern A: 3.97^a^, movement pattern B: 3.51^a^, movement pattern C: 3.97^a^
Kristjansson 2010, case control	Non-traumatic neck pain, > 6 months, < 6 years, **VAS_max_ 6.7 (2.6), 7♂11♀, 38.0 (8.3), NR, NR, NR. WAD (II), NR, VAS_max_ 8.0 (1.4), 2♂16♀, 35.5 (11.9), NR, NR, NR	10♂ 8♀, 32.2 (10.9), NR, NR, NR	Research assistant, NR, yes	Yes, follow a target, yes, yes, sitting, NR, NR, NR, NR, no	3-Space Fastrak system, NR, NR, − 0.89 mm to 1.03 mm, 0.78 (0.74–0.81), NR	Non-traumatic NP: Easy (mm) 2.06 (0.52). Medium (mm) 2.70 (0.88). Difficult (mm) 3.42 (1.30)	WAD (II): Easy 2.52 (0.78). Medium (mm) 3.45 (1.45). Difficult (mm) 4.09 (1.51)	HC: Easy (mm) 1.78 (0.33)^b^. Medium (mm) 2.17 (0.44)^ab^. Difficult (mm) 2.64 (0.52)^ab^
Meisingset 2015, case control	NP, > 2 weeks, NPRS 4.6 (1.4), 20♂, 55♀, 43.1 (12.9), 24.9 (4.7), private physiotherapy clinic and specialised neck and back pain clinic at university hospital, NR	43♂, 48♀, 40.8 (13.8), 25 (3.5), university hospital, NR	Physiotherapist, well trained, NR	Unclear, AROM, JPE, follow a motion pattern, unclear, yes, sitting, back support, fixed to back support, end range, self-selected, yes (JPE test)	Electromagnetic motion tracker system (Polhemus, Inc, Colchester, Vermont, USA), 240 Hz, NR, NR, NR, NR	NP. Head reposition: 5.6 (2.2), figure of eight low speed (cm): 3.4 (1.3), figure of eight high speed (cm): 4.4 (1.8), figure of eight standing low speed (cm): 2.9 (0.9). Fly easy (cm): 2.2 (0.9), fly medium (cm): 3.1 (0.9)		HC. Head reposition: 5.1 (1.9), figure of eight low speed (cm): 3.8 (1.4), figure of eight high speed (cm): 5.2^a^ (1.9), figure of eight standing low speed (cm): 3.3 (1.5). Fly easy (cm): 2.5^a^ (0.9), fly medium (cm): 3.3 (1.5)
Woodhouse 2008, case control	WAD I and II, > 6 months, NPRS 5.60 (2.49), 22♂, 34♀, 38.19 (10.8), NR (NR), referred, NR. CNP, > 6 months, NPRS 3.84 (− 1.74), 19♂, 38♀, 43.7 (12.6), NR (0), physiotherapists and general practitioners, NR	29♂, 28♀, 38.2 (10.9), NR (NR), different, NR	NR, NR, no	NR, AROM, NR, yes, sitting, NR, end range, NR, eyes closed	3-Space Fastrak, 120 Hz, reference, NR, NR, NR	WAD, neutral task: 3.35 (1.6)	CNP, neutral task: 3.17 (1.1)	HC, neutral task: 2.86 (1.2)

*Abbreviations*: *AROM* active range of motion, BMI body mass index, *CNP* chronic neck pain, *CE* constant error, *F/E* flexion/extension, *HC* healthy controls, *HRE* head repositioning error, *ICC* intraclass correlation coefficients, *JPE* joint position error, *LF* lateral flexion, *LOA* limits of agreement, *LR* left rotation, *NA* not applicable, *NPRS* numeric pain rating scale, *NR* not reported, *R* rotation, *RR* right rotation, *SD* standard deviation, *SEM* standard error of measurement, *VAS* visual analog scale, *WAD* whiplash-associated disorder

*Is test position in sitting, standing, with back support? Is the test subject fixed to the back support? Test to end range or pain limit? Movement speed self-selected, fast as possible or fixed? Is the test subject blindfolded?

**Participants were asked about maximum pain level

^a^Significant difference neck pain group I vs HC (*p* > 0,05)

^b^Significant difference neck pain group II vs HC (*p* > 0,05)

^c^Significant difference neck pain group I vs II (*p* > 0,05). ≈ Read from a graph

### Posture

In five studies [43, 47, 56, 58], measures of posture were assessed. The characteristics of the studies are described in Table 6. In three of these studies [43, 58], a working task of typing/computer work was assessed. In another one of these studies [47], the postural task of habitual sitting posture and perceived ‘good’ posture were evaluated, and in the remaining study [57], habitual standing posture was measured. ‘Sagittal plane angle of head tilt’ was the only parameter that was reported in all five studies. In two studies [44, 74], no difference between the measured angles in the different groups was found, and in the three other studies, between one [57] and two angles [47, 58] differed between the groups. However, none of the differences were consistent across the studies.

**Table 6:** Description of studies measuring neck posture

First author, year, design	Study population		Testing circumstances		Device	Comparison of posture	
	Type of neck pain, pain duration, pain intensity, sex (♂,♀), mean age (SD), mean BMI (SD), recruitment, occupation	Controls’ sex (♂,♀), mean age (SD), mean BMI (SD), recruitment, occupation	Examiners professional background, training, blinding	Instruction standardised, type of test, training, repeated, restrictions applied*	Type, sample rate (Hz), measurement error, LOA, ICC, SEM	Neck pain group I, type degrees (SD)	Healthy controls type degrees (SD)
Arvidsson et al. 2006, cohort	Neck-shoulder disorders, NR, NR, 0♂ 13♀, 38 (NR), NR, workplace, air traffic control	0♂, 11♀, 35 (NR), NR (NR), workplace, air traffic control	Physical therapist, NR, NR	NA, ordinary work, NA, no, sitting, NA, NA, NA, NR	Inclinometry, 20 Hz, NR, NR, NR, NR	Neck-shoulder disorders, 95th–5th, neck: 44 (9), head: 39 (8)	HC, 95th–5th, neck: 42 (10), head: 34 (7)
Edmondston 2007, case control	Postural neck pain, 5.2 (4.28) years, VAS 48.3 (14.81), 10♂11♀, 29.0 (7.36), NR,advertisement, NR	10♂ 12♀, 25.7 (5.95), NR, advertising, NR	Experienced physiotherapist, NR, NR	Yes, habitual sitting posture, perceived good posture and JPE, yes, yes, sitting, no, NA, NA, blindfolded	Three-dimensional optical motion analysis system (PEAK Performance Technologies Inc., Centennial, CO, USA), 50 Hz, NR, NR, NR, NR	Postural neck pain, habitual sitting posture, cervicothoracic: 158.0 (5.75), head tilt: 64.8 (5.41), head protraction: 170.0 (8.24), shoulder protraction: 11.7 (4.74), perceived ‘good’ posture, cervicothoracic: 153.6 (5.87), head tilt: 59.8 (7.00), head protraction: 169.6 (7.25), shoulder protraction: 12.7 (5.27)	HC, habitual sitting posture, cervicothoracic: 157.0 (6.22), head tilt: 68.0 (7.26), head protraction: 166.6 (6.82), shoulder protraction: 11.9 (6.33), perceived ‘good’ posture, cervicothoracic: 151.5 (4.91), head tilt: 64.4 (7.78)^a^, head protraction: 165.0 (7.85)^a^, shoulder protraction: 11.8 (6.08)
Silva 2009, case control	CNP, > 6 month, < 30 years, NPRS 5.6 (2.1), 6♂34♀, 50.2 (7.9), NR, referred by a physician for physiotherapy because of NP at the Hospital da Prelada, NR	6♂ 34♀, 50.2 (7.9), NR, general population, NR	NR, NR, yes	Yes, postural, no, no, standing, no, habitual, no movement, no	Video camera setup and APAS software, 25 Hz, NR, NR, NR, NR	CNP, habitual, C7, tragus, horizontal: 45.4 (6.8), tragus, eye, horizontal: 21.0 (6.4), Right ear, left ear, horizontal: 2.3 (1.8)	HC, habitual, C7, tragus, horizontal: 48.6^a^ (7.1), tragus, eye, horizontal: 18.8 (7.7), right ear, left ear, horizontal: 1.8 (1.5)
Szeto 2005, case control	NP, > 3 months, NR, 0♂21♀, 36 (4.6), NR, NR, office workers	0♂ 17♀, NR, NR, NR, office workers	NR, NR, NR	NR, 1 h typing, NR, no, sitting, no, habitual, no movement, no	Vicon 370, 60 Hz, 0.94 mm, NR, NR, NR	NP, 1 h typing, Head F/E: 67.59 (10.8), Head LF: − 1.14 (1.8), head R: 1.78 (2.4)	HC, 1 h typing, head F/E: 63.74 (12.9), head LF: − 2.67^a^ (2.2), head R: 4.23^a^ (2.5)
Xie Yf 2018, case control	Non-specific neck pain, > 3 months, NPRS 4.9 (1.8), 8♂11♀, 24.4, NR, poster advertisements in the local universities, NR	7♂ 11♀, 23.2 (3.3), NR, poster advertisements in the local universities, NR	NR, NR, NR	Unclear, taping on 1: smart phone one hand 2: both hands 3: computer, yes, no, sitting with back support, no, NA, NA,	Inertial measurement unit motion sensors (MyoMotion Clinical, Noraxon U.S.A. Inc.), 1500, 1°, NR, NR, NR	Non-specific NP, one hand F/E: ≈ 24.9 (6.6), R: ≈ RR 3.4 ( 6.3), LF: ≈ RLF 2.7 (5.8), both hands F/E: 28.8 (5.9), R: ≈ LR 0.4 (4.3), LF: ≈ RLF 3.1 (5.3), computer typing F/E: ≈ 4.5 (11.7), R: ≈ LR 0.4 (1.4), LF: ≈ RLF 1.2 (4.1)	HC, one hand, F/E: ≈ 25.8 (8.8), R: ≈ RR 0.4 (4.1), LF: ≈ LLF 0.8 (5.4), both hands F/E: ≈ 29.3 (6.7), R: ≈ LR 1.6 (3.1), LF: ≈ RLF 0.5 (4,3), computer typing F/E: ≈ 2.9 (6.8), R: ≈ RR 0.4 (6.6), LF: ≈RLF 1.6 (2.3)

*Abbreviations*: *BMI* body mass index, *CNP* chronic neck pain, *HC* healthy controls, *ICC* intraclass correlation coefficients, *JPE* joint positioning error, *LOA* limits of agreement, *NA* not applicable, *NP* neck pain, *NPRS* numeric pain rating scale, *NR* not reported, *RR* right rotation, *SD* standard deviation, *SEM* standard error of measurement, *VAS* visual analog scale

*Is test position in sitting, standing, with back support? Is the test subject fixed to the back support? Test to end range or pain limit? Movement speed self-selected, fast as possible or fixed? Is the test subject blindfolded?

^a^Significant difference neck pain group I vs HC (*p* > 0,05)

^b^Significant difference neck pain group II vs HC (*p* > 0,05)

^c^Significant difference neck pain group I vs II (p > 0,05). ≈ Red from a graph

## Discussion

Regardless of definition, people with varying types of neck pain have reduced active range of motion, reduced movement speed, and impaired head repositioning accuracy when compared with people without neck pain. However, due to lack of consistency in measurement parameters and variation in the postural tasks examined, it was not possible to quantify differences between people with and without neck pain for several of the included measures. We found substantial heterogeneity in the included studies regarding types of patients, types of measurements, and types of technology, and many studies had poor reporting, which resulted in high risk of bias.

Consequently, results of this review must be interpreted with caution. Firstly, study populations were poorly described, i.e. in 15 studies, the age and sex distribution were uneven across groups; secondly, the description of the neck pain groups was heterogeneous with eight different definitions of non-specific neck pain, and only five studies adequately reported the power calculation for their sample size. Furthermore, we found a general lack of description of the examiners’ background and training, which may influence patient handling and application of measurement devices as most of the measurement devices are dependent on the examiners’ ability to palpate landmarks on the subject, which is a challenge even for experienced clinicians [70, 71]. Also, blinding of assessors is mostly not reported in the articles, which may be a concern because body language and communication generally may be affected if the assessors have knowledge of clinical information and previous test results. Lastly, the variation in test methods and measurement parameters was large, making it unfeasible to do meta-analyses. This heterogeneity in test condition is most likely contributing to the large degree of variation in the measured values for people without neck pain. For example, cervical flexion ranged between 32 [29] and 65.3° [50] and extension between 28 [29] and 79.4° [51].

The complexity of delivering measurements of active range of motion has been the subject of several systematic reviews [36, 72, 73]. Williams et al. [72] concluded that the simple non-electronic measurement devices were more reliable when measuring cervical range of motion when compared with more sophisticated electronic devices, whereas Micheils et al. [73] found that electronic devices were more reliable and valid in assessing motion patterns (the fly) and that neck pain patients had a greater degree of error when compared with people without neck pain. We found conflicting results with three studies having a greater degree of error and one with a lesser degree of error for the neck pain patients. Lastly, de Vries et al. [36] reviewed the literature dealing with joint positioning sense in people with neck pain and people without neck pain and concluded that joint positioning error was greater for people with neck pain, which corresponds with our findings, although they also included non-electronic measurement devices.

To our knowledge, this is the first systematic review combining studies dealing with measurements of movement in people with different types of neck pain and different types of movement and postural impairments. We adhered to the criteria adapted from the Cochrane diagnostic studies handbook and reported our work according to the PRISMA guidelines. We searched literature in PubMed and Embase databases and closely scrutinised the reference list of the included studies. Due to the development of technologies and the ability to obtain more than one measurement in one test, we chose only to include studies using an electronic measurement device. The same argument was used in the choice of inclusion period for the studies.

We assessed the quality of the studies by adapting the QUADAS 2 tool for our purposes. QUADAS 2 is designed to assess diagnostic studies [39, 74]. According to their criteria, the case-control design has inherited risk of bias. However, when looking for how motion parameters differ between people with and without neck pain, the case-control design is feasible, but here, the inclusion of cases and controls is a potential source of bias. We took this into account in the assessment of the quality of the studies by assessing the way in which sample sizes were estimated, the recruitment strategy, and the description of both cases and controls. Another aspect we modified was the blinding of assessor to knowledge of clinical information and previous test results. Lastly, we included all studies regardless of risk of bias because we wanted to describe the body of literature comprehensively.

Clinical implications of this review include the potential for measures of movement impairments to be used as a tool for subgrouping and as a guiding intervention for neck pain patients. Targeting interventions to movement impairments may result in better outcomes of treatment. For example, Meisingset et al. [75] showed that improving postural control and neck flexibility was associated with a decrease in neck pain over a 2-month course of physiotherapy, whereas this was not the case for movement speed and positioning sense [76]. Importantly, however, targeting movement impairments alone is unlikely to be the ‘magic bullet’ in treating people with neck pain because of the potential underlying psychological and social factors, but it may still be a valuable addition [77]. Cross-sectional studies included in this literature review do not give insight into the underlying reasons for movement impairments, but they do provide evidence for their presence in people with neck pain.

Future research into movement impairments should apply uniform test methods and measurement parameters, and a set of consensus guidelines would greatly improve the comparison of studies. In addition, there is a need to assess the clinical usefulness of these measures in longitudinal cohort studies and as outcome measures in randomised clinical trials. Finally, wearable sensors built into headphones, smartphones, wristbands, patches, or clothes may provide new possibilities for investigating both the underlying factors involved in movement impairments and the influence of these impairments on activities of daily living.

## Conclusion

People with varying types of neck pain have reduced active range of motion, reduced movement speed, and impaired head repositioning accuracy when compared with people without neck pain. Due to poor and inconsistent reporting regarding test methods, test subjects, blinding of examiners, and examiner background and training, these results should be interpreted with caution. Longitudinal studies are necessary to investigate the underlying factors for movement impairments and their potential to guide clinical interventions.

## Additional files

Additional file 1: PRISMA checklist. (DOCX 110 kb)

Additional file 2: Search methods for identification of studies. (DOC 62 kb)

Additional file 3: Excluded Studies. (DOCX 28 kb)

## Data Availability

All data generated or analysed during this study are included in this published article and its additional files.

## Abbreviations

BBH: Bue Bonderup Hesby
GRRAS: Guidelines for Reporting Reliability and Agreement Studies
HR: Hanne Rasmussen
JH: Jan Hartvigsen
PK: Per Kjaer
QUADAS: Quality assessment of studies of diagnostic accuracy included in systematic reviews
RoM: Active range of motion
WAD: Whiplash-associated disorder
