# Supplementary material for: Electronic measures of movement impairment, repositioning, and posture in people with and without neck pain—a systematic review
Source: Syst Rev. 2019 Aug 27;8:220. doi: 10.1186/s13643-019-1125-2 (PMC6710866; doi:10.1186/s13643-019-1125-2)
Supplement: Supplementary file 1 — PRISMA checklist. (DOCX 110 kb) [file 13643_2019_1125_MOESM1_ESM.docx]

# Additional File 3: Excluded Studies

## Reasons for exclusion of full text studies

1. Measuring device was not electronic: [1-16]
2. Exclusion of pain group or no actual pain in pain group: [7, 13, 17-26]
3. Control group not pain-free or lack of description of study participants:[27-33]
4. Lack of description of measuring method/device: [3, 34]
5. Motion test not of interest: [35-41]
6. Data not presented to inform inclusion criteria for the review: [42-45]
7. Age group <18 years:[46, 47]
8. Passive range of motion test before active: [48]
9. Conference abstract only: [49-53]

## References

1. Subbarayalu AV: **Measurement of craniovertebral angle by the Modified Head Posture Spinal Curvature Instrument: A reliability and validity study**. *Physiotherapy theory and practice* 2016, **32**(2):144-152.

2. Mani S, Sharma S, Omar B, Ahmad K, Muniandy Y, Singh DKA: **Quantitative measurements of forward head posture in a clinical settings: a technical feasibility study**. *European Journal of Physiotherapy* 2017, **19**(3):119-123.

3. Elsig S, Luomajoki H, Sattelmayer M, Taeymans J, Tal-Akabi A, Hilfiker R: **Sensorimotor tests, such as movement control and laterality judgment accuracy, in persons with recurrent neck pain and controls. A case-control study**. *Manual Therapy* 2014, **19**(6):555-561.

4. Fernandez-Perez AM, Villaverde-Gutierrez C, Mora-Sanchez A, Alonso-Blanco C, Sterling M, Fernandez-de-Las-Penas C: **Muscle trigger points, pressure pain threshold, and cervical range of motion in patients with high level of disability related to acute whiplash injury**. *The Journal of orthopaedic and sports physical therapy* 2012, **42**(7):634-641.

5. Fletcher JP, Bandy WD: **Intrarater reliability of CROM measurement of cervical spine active range of motion in persons with and without neck pain**. *The Journal of orthopaedic and sports physical therapy* 2008, **38**(10):640-645.

6. Kaale BR, Krakenes J, Albrektsen G, Wester K: **Active range of motion as an indicator for ligament and membrane lesions in the upper cervical spine after a whiplash trauma**. *Journal of neurotrauma* 2007, **24**(4):713-721.

7. Lee H, Nicholson LL, Adams RD: **Cervical range of motion associations with subclinical neck pain**. *Spine* 2004, **29**(1):33-40.

8. Lee H, Nicholson LL, Adams RD: **Neck muscle endurance, self-report, and range of motion data from subjects with treated and untreated neck pain**. *Journal of manipulative and physiological therapeutics* 2005, **28**(1):25-32.

9. Nilsson BM, Soderlund A: **Head posture in patients with whiplash-associated disorders and the measurement method's reliability - A comparison to healthy subjects**. *Advances in Physiotherapy* 2005, **7**(1):13-19.

10. Palmgren PJ, Andreasson D, Eriksson M, Hagglund A: **Cervicocephalic kinesthetic sensibility and postural balance in patients with nontraumatic chronic neck pain - A pilot study**. *Chiropractic and Osteopathy* 2009, **17**(6).

11. Peolsson A, Ludvigsson ML, Wibault J, Dedering A, Peterson G: **Function in patients with cervical radiculopathy or chronic whiplash-associated disorders compared with healthy volunteers**. *Journal of manipulative and physiological therapeutics* 2014, **37**(4):211-218.

12. Shahidi B, Johnson CL, Curran-Everett D, Maluf KS: **Reliability and group differences in quantitative cervicothoracic measures among individuals with and without chronic neck pain**. *BMC musculoskeletal disorders* 2012, **13**:215.

13. Wibault J, Vaillant J, Vuillerme N, Dedering A, Peolsson A: **Using the cervical range of motion (CROM) device to assess head repositioning accuracy in individuals with cervical radiculopathy in comparison to neck- healthy individuals**. *Manual therapy* 2013, **18**(5):403-409.

14. Yip CH, Chiu TT, Poon AT: **The relationship between head posture and severity and disability of patients with neck pain**. *Manual therapy* 2008, **13**(2):148-154.

15. Cheung CH, Shum ST, Tang SF, Yau PC, Chiu TT: **The correlation between craniovertebral angle, backpack weights, and disability due to neck pain in adolescents**. *Journal of back and musculoskeletal rehabilitation* 2010, **23**(3):129-136.

16. Ghamkhar L, Kahlaee AH, Nourbakhsh MR, Ahmadi A, Arab AM: **Relationship Between Proprioception and Endurance Functionality of the Cervical Flexor Muscles in Chronic Neck Pain and Asymptomatic Participants**. *J Manipulative Physiol Ther* 2018, **41**(2):129-136.

17. Basteris A, Pedler A, Sterling M: **Evaluating the neck joint position sense error with a standard computer and a webcam**. *Manual Therapy* 2016, **26**:231-234.

18. Inokuchi H, Tojima M, Mano H, Ishikawa Y, Ogata N, Haga N: **Neck range of motion measurements using a new three-dimensional motion analysis system: validity and repeatability**. *European Spine Journal* 2015, **24**(12):2807-2815.

19. Lee H, Nicholson LL, Adams RD, Bae SS: **Proprioception and rotation range sensitization associated with subclinical neck pain**. *Spine* 2005, **30**(3):E60-67.

20. Teng CC, Chai H, Lai DM, Wang SF: **Cervicocephalic kinesthetic sensibility in young and middle-aged adults with or without a history of mild neck pain**. *Manual therapy* 2007, **12**(1):22-28.

21. Uremovic M, Bosnjak-Pasic M, Sekelj-Kauzlaric K, Lisak M, Demarin V: **Evaluation of proprioception by a standard instrument for measurement of cervical spine movement - Cervical measurement system**. *Acta Clinica Croatica* 2005, **44**(4):335-341.

22. Uremovic M, Cvijetic S, Pasic MB, Seric V, Vidrih B, Demarin V: **Impairment of proprioception after whiplash injury**. *Collegium antropologicum* 2007, **31**(3):823-827.

23. Van den Oord MH, De Loose V, Sluiter JK, Frings-Dresen MH: **Neck strength, position sense, and motion in military helicopter crew with and without neck pain**. *Aviation, space, and environmental medicine* 2010, **81**(1):46-51.

24. Vikne H, Bakke ES, Liestol K, Engen SR, Vollestad N: **Muscle activity and head kinematics in unconstrained movements in subjects with chronic neck pain; cervical motor dysfunction or low exertion motor output?** *BMC musculoskeletal disorders* 2013, **14**:314.

25. Nagai T, Abt JP, Sell TC, Clark NC, Smalley BW, Wirt MD, Lephart SM: **Neck proprioception, strength, flexibility, and posture in pilots with and without neck pain history**. *Aviation, space, and environmental medicine* 2014, **85**(5):529-535.

26. Nourollahi-Darabad M, Mazloumi A, Saraji GN, Afshari D, Foroushani AR: **Full shift assessment of back and head postures in overhead crane operators with and without symptoms**. *Journal of occupational health* 2018, **60**(1):46-54.

27. Bush TR, Vorro J, Alderink G, Gorbis S, Li M, Leitkam S: **Relating a manual medicine diagnostic test of cervical motion function to specific three-dimensional kinematic variables**. *International Journal of Osteopathic Medicine* 2010, **13**(2):48-55.

28. De Loose V, Van den Oord M, Burnotte F, Van Tiggelen D, Stevens V, Cagnie B, Danneels L, Witvrouw E: **Functional assessment of the cervical spine in F-16 pilots with and without neck pain**. *Aviation, space, and environmental medicine* 2009, **80**(5):477-481.

29. Kauther MD: **Answer to the letter to the editor of Hilla Sarig Bahat entitled "do these large numbers contrast multiple smaller-number prior studies?": Re: Kauther et al. 2012, Cervical range of motion and strength in 4,293 young male adults with chronic neck pain. European Spine Journal 21:1522-1527**. *European Spine Journal* 2013, **22**(5):1193-1194.

30. Kauther MD, Piotrowski M, Hussmann B, Lendemans S, Wedemeyer C: **Cervical range of motion and strength in 4,293 young male adults with chronic neck pain**. *European spine journal : official publication of the European Spine Society, the European Spinal Deformity Society, and the European Section of the Cervical Spine Research Society* 2012, **21**(8):1522-1527.

31. Lee HY, Wang JD, Yao G, Wang SF: **Association between cervicocephalic kinesthetic sensibility and frequency of subclinical neck pain**. *Manual therapy* 2008, **13**(5):419-425.

32. Roren A, Mayoux-Benhamou MA, Fayad F, Poiraudeau S, Lantz D, Revel M: **Comparison of visual and ultrasound based techniques to measure head repositioning in healthy and neck-pain subjects**. *Manual therapy* 2009, **14**(3):270-277.

33. Bergman GJ, Knoester B, Assink N, Dijkstra PU, Winters JCJS: **Variation in the cervical range of motion over time measured by the “flock of birds” electromagnetic tracking system**. 2005, **30**(6):650-654.

34. Pinsault N, Vuillerme N, Pavan P: **Cervicocephalic relocation test to the neutral head position: assessment in bilateral labyrinthine-defective and chronic, nontraumatic neck pain patients**. *Archives of physical medicine and rehabilitation* 2008, **89**(12):2375-2378.

35. Lopez-de-Uralde-Villanueva I, Acuyo-Osorio M, Prieto-Aldana M, La Touche R: **Reliability and minimal detectable change of a modified passive neck flexion test in patients with chronic nonspecific neck pain and asymptomatic subjects**. *Musculoskeletal Science and Practice* 2017, **28**:10-17.

36. Takasaki H, Treleaven J, Johnston V, Van den Hoorn W, Rakotonirainy A, Jull G: **A description of neck motor performance, neck pain, fatigue, and mental effort while driving in a sample with chronic whiplash-associated disorders**. *American journal of physical medicine & rehabilitation / Association of Academic Physiatrists* 2014, **93**(8):665-674.

37. Treleaven J, Takasaki H: **High variability of the subjective visual vertical test of vertical perception, in some people with neck pain - Should this be a standard measure of cervical proprioception?** *Manual Therapy* 2015, **20**(1):183-188.

38. Tsang SM, Szeto GP, Lee RY: **Altered spinal kinematics and muscle recruitment pattern of the cervical and thoracic spine in people with chronic neck pain during functional task**. *Journal of electromyography and kinesiology : official journal of the International Society of Electrophysiological Kinesiology* 2014, **24**(1):104-113.

39. Oddsdottir GL, Kristjansson E, Gislason MK: **Sincerity of effort versus feigned movement control of the cervical spine in patients with whiplash-associated disorders and asymptomatic persons: a case-control study**. *Physiother Theory Pract* 2015, **31**(6):403-409.

40. Tsang SMH, Szeto GPY, Lee RYW: **Relationship between neck acceleration and muscle activation in people with chronic neck pain: Implications for functional disability**. *Clinical Biomechanics* 2016, **35**:27-36.

41. Lascurain-Aguirrebena I, Newham DJ, Galarraga-Gallastegui B, Critchley DJ: **Differences in neck surface electromyography, kinematics and pain occurrence during physiological neck movements between neck pain and asymptomatic participants. A cross-sectional study**. *Clinical Biomechanics* 2018, **57**:1-9.

42. Niederer D, Vogt L, Wilke J, Rickert M, Banzer W: **Age-related cutoffs for cervical movement behaviour to distinguish chronic idiopathic neck pain patients from unimpaired subjects**. *European Spine Journal* 2015, **24**(3):493-502.

43. Treleaven J, Jull G, LowChoy N: **The relationship of cervical joint position error to balance and eye movement disturbances in persistent whiplash**. *Manual therapy* 2006, **11**(2):99-106.

44. Woodhouse A, Stavdahl O, Vasseljen O: **Irregular head movement patterns in whiplash patients during a trajectory task**. *Experimental brain research* 2010, **201**(2):261-270.

45. Dunleavy K, Neil J, Tallon A, Adamo DE: **Reliability and validity of cervical position measurements in individuals with and without chronic neck pain**. *Journal of Manual and Manipulative Therapy* 2015, **23**(4):188-196.

46. Park KN, Kwon OY, Ha SM, Kim SJ, Choi HJ, Weon JH: **Comparison of electromyographic activity and range of neck motion in violin students with and without neck pain during playing**. *Medical problems of performing artists* 2012, **27**(4):188-192.

47. Straker LM, O'Sullivan PB, Smith AJ, Perry MC: **Relationships between prolonged neck/shoulder pain and sitting spinal posture in male and female adolescents**. *Manual therapy* 2009, **14**(3):321-329.

48. Rutledge B, Bush TR, Vorro J, Li M, DeStefano L, Gorbis S, Francisco T, Seffinger M: **Differences in human cervical spine kinematics for active and passive motions of symptomatic and asymptomatic subject groups**. *Journal of applied biomechanics* 2013, **29**(5):543-553.

49. De Pauw R, Coppieters I, Danneels L, Cagnie B: **Influence of kinesiophobia and symptoms of central sensitization on motor behaviour in patients with chronic neck pain**. *Manual Therapy* 2016, **25**:e89.

50. Lin CL, Lin CF: **Comparison of neck position sense and balance control between neck pain patients and pain-free controls**. *Spine Journal* 2016, **16 (10 Supplement 1)**:S384.

51. Reid S, Portelli A: **Cervical proprioception in young adults with and without neck pain, who spend prolonged time on mobile devices: An observational study**. *Manual Therapy* 2016, **25**:e86-e87.

52. Snodgrass S, Cooper R, Edwards S, Moghaddas D, Blyton S, De Zoete R, Rivett D: **Altered movement strategies during functional tasks in individuals with chronic idiopathic neck pain**. *Manual therapy* 2016, **25**:e68-e69.

53. Tsang SMH, Szeto GPY, Lee RYW: **Spinal movement coordination and muscular activity in adults with chronic mechanical neck pain**. *Hong Kong Physiotherapy Journal* 2011, **29 (2)**:99.
