# Supplementary material for: Electronic measures of movement impairment, repositioning, and posture in people with and without neck pain—a systematic review
Source: Syst Rev. 2019 Aug 27;8:220. doi: 10.1186/s13643-019-1125-2 (PMC6710866; doi:10.1186/s13643-019-1125-2)
Supplement: Supplementary file 3 — Excluded Studies. (DOCX 28 kb) [file 13643_2019_1125_MOESM3_ESM.docx]

# Additional file 2

# Search methods for identification of studies

Two review authors and research librarians searched the database MEDLINE and EMBASE for literature on the basis of Table 1.

Two review authors independently selected the articles; first the authors screened the titles for relevance. Selected articles then had the abstract screened. Remaining articles were read in full text and selected based on the inclusion and exclusion criteria. Uncertainties during the first two steps led to the article being included in the next step. Disagreements between authors were discussed and consensus sought. Continued disagreement was resolved by a third rater.

The rules are given in Table 2.

Table 1.

(pub med)

| Diagnosis [neck pain/classification](http://www.ncbi.nlm.nih.gov/pubmed/17070722) | | | Measurement |
| --- | --- | --- | --- |
| 1.  neck pain [mesh]  neck pain [ti/ab]  neck ache [ti/ab]  cervical pain [ti/ab]  cervical pains [ti/ab]  cervicodynia [ti/ab]  cervicalgia [ti/ab]  whiplash injuries [mesh]  whiplash injuries[ti/ab]  1.  ((((((((((whiplash injuries[ti/ab]) OR whiplash injuries [mesh]) OR cervicalgia [ti/ab]) OR cervicodynia [ti/ab]) OR neckaches [ti/ab]) OR neckache [ti/ab]) OR cervical pains [ti/ab]) OR cervical pain [ti/ab]) OR neck ache [ti/ab]) OR neck pain [ti/ab]) OR neck pain [mesh] | | | 6.  proprioception [mesh]  proprioception [ti/ab]  motor control [ti/ab]  kinesthetic [ti/ab]  kinematics[mesh]  kinematics [ti/ab]  posture [mesh]  posture[ti/ab]  postures[ti/ab]  range of motion, articular [mesh]  range of motion[ti/ab]  passive range of motion[ti/ab]  6.  ((((((((((Proprioception [mesh]) OR Proprioception [ti/ab]) OR motor control [ti/ab]) OR Kinesthetic [ti/ab]) OR Kinematics[mesh]) OR kinematics [ti/ab]) OR posture [mesh]) OR postures[ti/ab]) OR Range of Motion, Articular [mesh]) OR Range of Motion[ti/ab]) OR Passive Range of Motion[ti/ab] |
| 2.  Radiculopathy[mesh]  radiculopathies[ti/ab]  cervical radiculopathies[ti/ab]  cervical radiculopathy[ti/ab]  nerve root disorders[ti/ab]  radiculitis[ti/ab]  nerve root Inflammation[ti/ab]  nerve root avulsion[ti/ab]  nerve root avulsions [ti/ab]  nerve root compression [ti/ab]  nerve root compressions [ti/ab]  2.  (((((((((((Nerve Root Compressions [ti/ab]) OR Nerve Root Compression [ti/ab]) OR Nerve Root Avulsions [ti/ab]) OR Nerve Root Avulsion[ti/ab]) OR Nerve Root Inflammations[ti/ab]) OR Nerve Root Inflammation[ti/ab]) OR Radiculitis[ti/ab]) OR Nerve Root Disorders[ti/ab]) OR Cervical Radiculopathy[ti/ab]) OR Cervical Radiculopathies[ti/ab]) OR Radiculopathies[ti/ab]) OR Radiculopathy[mesh] | And | 3.  neck [mesh]  neck [ti/ab]  cervical [mesh]  cervical [ti/ab]  cervical vertebrae [mesh]  cervical vertebrae [ti/ab]  3.  (((((Neck [mesh]) OR Neck [ti/ab]) OR Cervical [mesh]) OR Cervical [ti/ab]) OR Cervical Vertebrae [mesh]) OR Cervical Vertebrae [ti/ab] |  |
| 2 and 3 = 4  4.  (((((((Neck [mesh]) OR Neck [ti/ab]) OR Cervical [mesh]) OR Cervical [ti/ab]) OR Cervical Vertebrae [mesh]) OR Cervical Vertebrae [ti/ab])) AND ((((((((((((Nerve Root Compressions [ti/ab]) OR Nerve Root Compression [ti/ab]) OR Nerve Root Avulsions [ti/ab]) OR Nerve Root Avulsion[ti/ab]) OR Nerve Root Inflammations[ti/ab]) OR Nerve Root Inflammation[ti/ab]) OR Radiculitis[ti/ab]) OR Nerve Root Disorders[ti/ab]) OR Cervical Radiculopathy[ti/ab]) OR Cervical Radiculopathies[ti/ab]) OR Radiculopathies[ti/ab]) OR Radiculopathy[mesh]) | | |  |
| 4 or 1 = 5  5.  (((((((((Neck [mesh]) OR Neck [ti/ab]) OR Cervical [mesh]) OR Cervical [ti/ab]) OR Cervical Vertebrae [mesh]) OR Cervical Vertebrae [ti/ab])) AND ((((((((((((Nerve Root Compressions [ti/ab]) OR Nerve Root Compression [ti/ab]) OR Nerve Root Avulsions [ti/ab]) OR Nerve Root Avulsion[ti/ab]) OR Nerve Root Inflammations[ti/ab]) OR Nerve Root Inflammation[ti/ab]) OR Radiculitis[ti/ab]) OR Nerve Root Disorders[ti/ab]) OR Cervical Radiculopathy[ti/ab]) OR Cervical Radiculopathies[ti/ab]) OR Radiculopathies[ti/ab]) OR Radiculopathy[mesh]))) OR (((((((((((whiplash injuries[ti/ab]) OR whiplash injuries [mesh]) OR cervicalgia [ti/ab]) OR cervicodynia [ti/ab]) OR neckaches [ti/ab]) OR neckache [ti/ab]) OR cervical pains [ti/ab]) OR cervical pain [ti/ab]) OR neck ache [ti/ab]) OR neck pain [ti/ab]) OR neck pain [mesh]) | | **5 and 6**  **(((((((((((Neck [mesh]) OR Neck [ti/ab]) OR Cervical [mesh]) OR Cervical [ti/ab]) OR Cervical Vertebrae [mesh]) OR Cervical Vertebrae [ti/ab])) AND ((((((((((((Nerve Root Compressions [ti/ab]) OR Nerve Root Compression [ti/ab]) OR Nerve Root Avulsions [ti/ab]) OR Nerve Root Avulsion[ti/ab]) OR Nerve Root Inflammations[ti/ab]) OR Nerve Root Inflammation[ti/ab]) OR Radiculitis[ti/ab]) OR Nerve Root Disorders[ti/ab]) OR Cervical Radiculopathy[ti/ab]) OR Cervical Radiculopathies[ti/ab]) OR Radiculopathies[ti/ab]) OR Radiculopathy[mesh]))) OR (((((((((((whiplash injuries[ti/ab]) OR whiplash injuries [mesh]) OR cervicalgia [ti/ab]) OR cervicodynia [ti/ab]) OR neckaches [ti/ab]) OR neckache [ti/ab]) OR cervical pains [ti/ab]) OR cervical pain [ti/ab]) OR neck ache [ti/ab]) OR neck pain [ti/ab]) OR neck pain [mesh]))) AND (((((((((((Proprioception [mesh]) OR Proprioception [ti/ab]) OR motor control [ti/ab]) OR Kinesthetic [ti/ab]) OR Kinematics[mesh]) OR kinematics [ti/ab]) OR posture [mesh]) OR postures[ti/ab]) OR Range of Motion, Articular [mesh]) OR Range of Motion[ti/ab]) OR Passive Range of Motion[ti/ab])** | |

Embase

| Diagnosis [neck pain/classification](http://www.ncbi.nlm.nih.gov/pubmed/17070722) | | | Measurement |
| --- | --- | --- | --- |
| **Keyword** :  cervicobrachial neuralgia  neck pain  cervical spondylosis  whiplash injury  **Abstrac**t:  cervicobrachial neuralgia  neck pain  cervicodynia  cervical spondylosis  neck ache  cervical pain  cervicalgia  whiplash injuries  whiplash associated disorder  cervical radiculopathies  cervical radiculopathy | | | Keyword:  body posture  proprioception  motor control  kinesthesia   \|  \| joint mobility/ or "range of motion"/ or "movement (physiology)"/ \| \| --- \| --- \|   kinematics  Abstract:   \|  \| proprioception \| \| --- \| --- \|   motor control  kinesthetic  kinematics  posture   \| ”range of motion”  body posture  joint mobility  ”movement (physiology)” \| \| --- \| |
| **Abstrac**t:  radiculopathies  nerve root disorders  radiculitis  nerve root inflammation  nerve root avulsion  nerve root avulsions  nerve root compression | **and** | **Abstrac**t:  neck  cervical  cervical vertebrae |  |
